# Redox Mechanisms upon the Lithiation of Wadsley–Roth Phases

**DOI:** 10.1021/acs.inorgchem.4c00603

**Published:** 2024-06-04

**Authors:** Muna Saber, Anton Van der Ven

**Affiliations:** † Department of Chemical Engineering, University of California, Santa Barbara, Santa Barbara, California 93106, United States; ‡ Materials Department, University of California, Santa Barbara, Santa Barbara, California 93106, United States

## Abstract

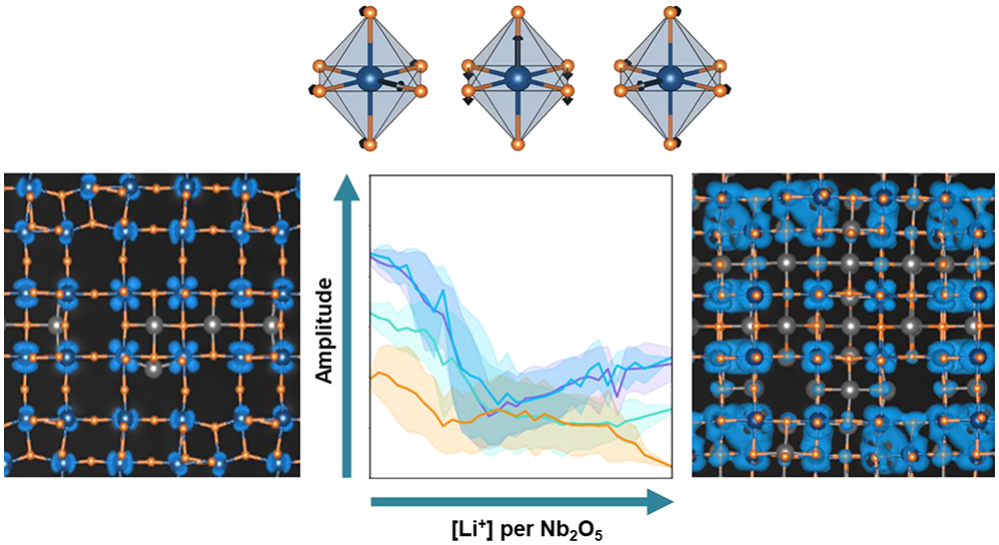

The Wadsley–Roth family of transition metal oxide phases are a promising class of anode materials for Li-ion batteries due to their open crystal structures and their ability to intercalate Li at high rates. Unfortunately, most early transition metal oxides that adopt a Wadsley–Roth crystal structure intercalate Li at voltages that are too high for most battery applications. First-principles electronic structure calculations are performed to elucidate redox mechanisms in Wadsley–Roth phases with the aim of determining how they depend on crystal structure. A comparative study of two very distinct polymorphs of Nb_2_O_5_ reveal two redox mechanisms: (i) an atom-centered redox mechanism at early stages of Li intercalation and (ii) a redox mechanism at intermediate to high Li concentrations involving the bonding orbitals of metal–metal dimers formed by edge-sharing Nb cations. Our study motivates several design principles to guide the development of new Wadsley–Roth phases with superior electrochemical properties.

## Abstract 2

A comprehensive ab initio study of the effect of Wadsley−Roth phase structural arrangement on polyhedral distortions, metal−metal bonding, and lithiation mechanisms.

## I Introduction

The high-power energy storage needs of automotive and aerospace applications require new electrode materials that can intercalate Li-ions at rates that exceed those of current carbon-based anodes. This has led to widespread interest in Wadsley–Roth phases as possible anode materials, as these phases are able to charge and discharge at exceptionally high rates within a voltage window of 2.5 and 0.75 V.^1−13^ Wadsley–Roth phases exhibit a rich diversity of crystal structures, consisting of infinitely long, rectangular blocks of corner-sharing metal–oxygen octahedra (MO_6_) that are joined together along “crystallographic shear planes” where the octahedra share edges.^14,15^ Their open structures allow for rapid Li insertion and removal, while the crystallographic shear planes give the blocks of corner-sharing octahedra some degree of rigidity. Promising Wadsley–Roth phases for anode applications include TiNb_2_O_7_,^1,3,16^ PNb_9_O_25_,^9,17−19^ and Nb_16_W_5_O_55_,^2^ among others.^20−23^ A significant impediment to the widespread use of Wadsley–Roth phases, however, is their high open-circuit voltage relative to Li, which reduces the voltage of a battery when they are used as the anode. As a result, there is a need to understand mechanisms of redox in Wadsley–Roth phases and how they can be manipulated through modifications of chemistry and crystal structure.

**Figure 1:**
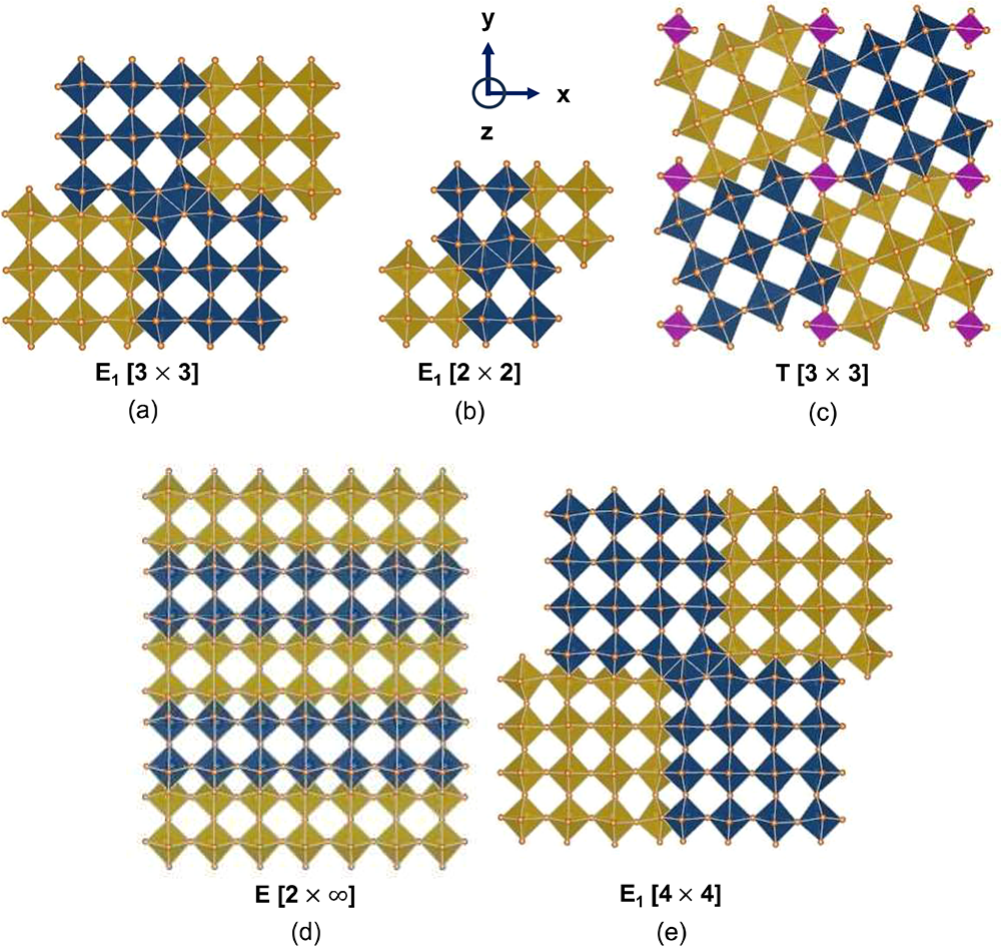
(a) The E_1_[3 × 3] Wadsley–Roth crystal structure adopted by TiNb_2_O_7_; (b) the TiO_2_-B structure, which was recently identified as an E_1_[2 × 2] Wadsley–Roth phase;^15^ (c) the T[3 × 3] structure of PNb_9_O_25_; (d) the E[2 × *∞*] Wadsley–Roth phase adopted by γ-Ta_2_O_5_; and (e) the E_1_[4 × 4] structure adopted by N-Nb_2_O_5_.

Figure 1 shows several Wadsley–Roth crystal structures. The original naming scheme of Wadsley–Roth phases introduced by Cava et al.^14^ was recently extended to be more descriptive and precise.^15^ The E_*iα*_[*n* × *m*] Wadsley–Roth phases, for example, consist of blocks of *n* × *m* corner-sharing octahedra joined together along shear boundaries that exclusively consist of edge-sharing octahedra. The subscript to E specifies the relative shift *i* in units of octahedral widths in the α direction (α = *x* or *y*). Figure 1a illustrates the E_1_[3 × 3] structure of TiNb_2_O_7_, an important commercial anode material^1,16,24−29^ (the shift direction α is redundant when *n* = *m*). The TiO_2_-B compound (Figure 1b), which is another promising high-rate anode material,^30,31^ was recently identified as an E_1_[2 × 2] Wadsley–Roth phase.^15^ The T[*n* × *m*] Wadsley–Roth phases differ from the E_*iα*_[*n* × *m*] structures in that they have tetrahedrally coordinated cations at the corners of the blocks. Figure 1c illustrates the T[3 × 3] structure of PNb_9_O_25_, which has phosphorus cations (P^5+^) residing in tetrahedral sites.^17,32^ Also shown in Figures 1d and e are two very different Wadsley–Roth structures that can be adopted by Nb_2_O_5_.^33^ The E[2 × *∞*] structure shown in Figure 1d, known as R-Nb_2_O_5_, consists of 2 × *∞* corner-sharing octahedral blocks^34−37^ and is the stable polymorph of γ-Ta_2_O_5_,^38,39^ while the E_1_[4 × 4] structure shown in Figure 1e consists of 4 × 4 blocks shifted relative to each other by one octahedral spacing and is an important polymorph of Nb_2_O_5_.^15^ This polymorph is also known as N-Nb_2_O_5_.^33,40−43^

**Figure 2:**
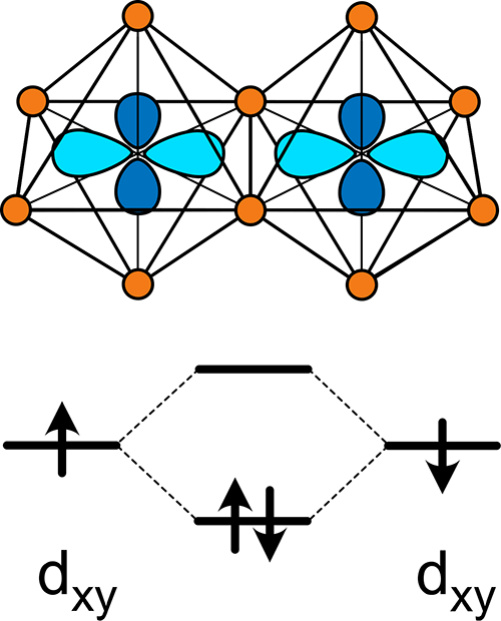
The t_2g_ orbitals, such as d_*xy*_, of early transition metal cations that reside in edge-sharing octahedra made of oxygen anions can hybridize to form metal–metal bonds that can host two electrons. Reproduced from Saber et al.^45^

The chemical and crystallographic diversity of Wadsley–Roth phases^14,15^ should offer a variety of ways to modify the voltage profile and Li transport properties of anodes made of early transition metal oxides. The design space is further enlarged by the availability of two qualitatively distinct redox mechanisms predicted to occur in Wadsley–Roth phases. The first is an atom-centric redox mechanism involving t_2g_ orbitals of early transition metals residing in corner-sharing octahedra^44^ that dominates at dilute Li concentrations. At less dilute Li concentrations, a different redox mechanism that relies on the formation of metal–metal dimers between edge-sharing MO_6_ octahedra was recently predicted to dominate in Li_*x*_TiNb_2_O_7_.^45^ The t_2g_ orbitals (i.e., d_*xy*_, d_*yz*_, and d_*xz*_) of early transition metal cations, having lobes that point between pairs of oxygen, can hybridize with neighboring t_2g_ orbitals to form bonding and antibonding states, as schematically illustrated in Figure 2. The bonding state, which has a lower energy than the unhybridized atom-centric t_2g_ orbitals, can host two electrons and thereby serve as a redox center to accommodate the electrons donated by Li. The metal-dimer redox mechanism requires edge-sharing octahedra containing early transition metals such as V, Nb and Mo, which are known to form metal–metal bonds in oxides.^46−49^ It is an unconventional redox mechanism that occurs on molecular-orbital-like states distributed over multiple ions within the solid.^48−51^

In this paper, we analyze redox mechanisms in Wadsley–Roth phases from first principles. We focus on two distinct polymorphs of Nb_2_O_5_ to identify crystallographic factors that affect redox mechanisms in Wadsley–Roth phases. Similar to TiNb_2_O_7_,^45^ two redox mechanisms are found to accommodate Li insertion into Nb_2_O_5_: an atom-centered charge compensation mechanism dominating at dilute Li concentrations and a redox mechanism that relies on the formation of metal–metal dimers dominating at more concentrated Li contents. The metal–dimer redox mechanism induces structural distortions that result in sizable changes in lattice parameters and unit cell shape. The insights of this study taken together with those generated in previous work^15,18,44,45,52^ suggest materials design principles with which the voltage of Wadsley–Roth phases can be tailored.

## II Methods

Density functional theory (DFT) calculations were performed using the Vienna Ab initio Simulation Package (VASP)^53−56^ within the approximation of Perdew, Burke, and Ernzerhof (PBE).^57^ Interactions between valence electrons and core electrons were treated with the projector augmented wave (PAW) method.^58,59^ All electronic structure calculations were performed spin-polarized and were initialized in the ferromagnetic state. A plane-wave energy cutoff of 650 eV and k-point grid with a reciprocal space discretization of 25 K-points per Å^–1^ were used. Lithium vacancy orderings in the E[2 × *∞*] and E_1_[4 × 4] Nb_2_O_5_ Wadsley–Roth phases were enumerated using the Clusters Approach to Statistical Mechanics (CASM) software package.^60−62^

## III Results

### A Formation Energies and Zero Kelvin Voltage Profiles

To establish the role of structure on the redox mechanisms in Wadsley–Roth phases, we analyze the results of first-principles electronic structure calculations performed on two very distinct Wadsley–Roth crystal structures having the same chemical composition: Nb_2_O_5_ in the E[2 × *∞*] and E_1_[4 × 4] structures. Lithium ions in Wadsley–Roth phases can occupy pyramidal sites coordinated by five oxygen ions and square planar window sites coordinated by four oxygen ions.^18,44,45,63^ The pyramidal sites reside along the shear boundaries, at the edges of the *n* × *m* blocks of corner-sharing octahedra, while the window sites reside within the *n* × *m* blocks. The formation energies of a large number of Li arrangements over the pyramidal and window sites of the E[2 × *∞*] and E_1_[4 × 4] forms of Nb_2_O_5_ were calculated with DFT-PBE.

**Figure 3:**
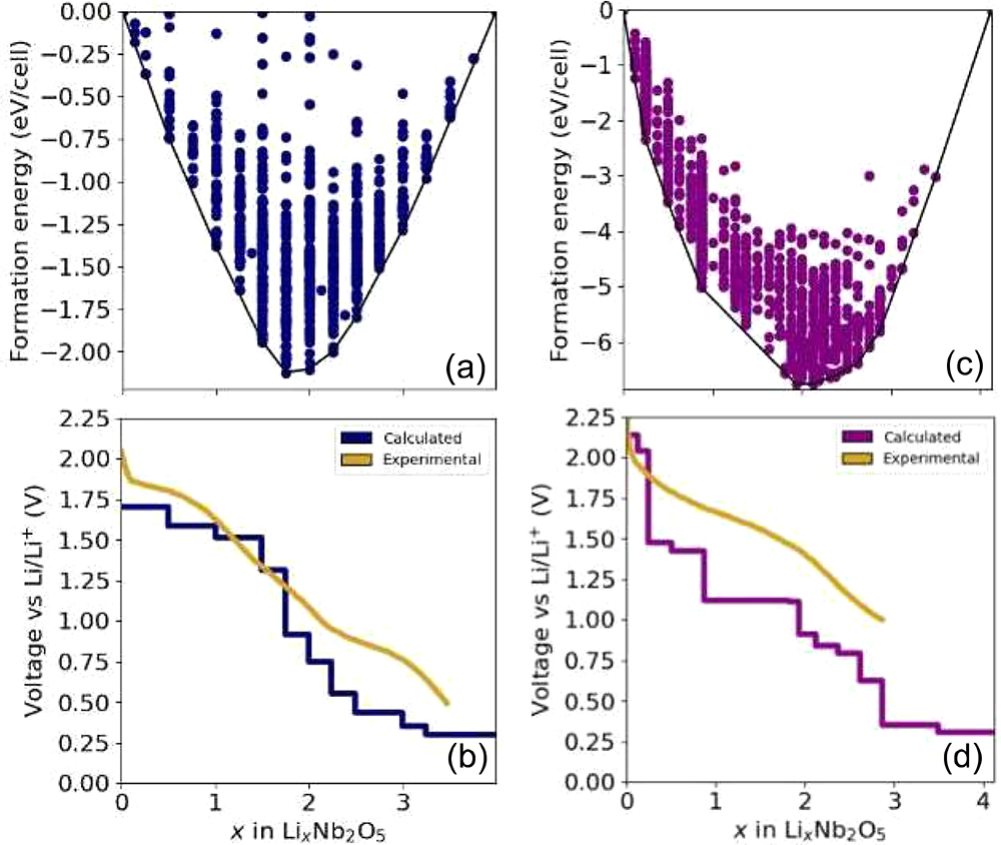
Formation energies of Li-vacancy orderings over the interstitial sites of the E[2 × *∞*] (a) and E_1_[4 × 4] (b) polymorphs of Nb_2_O_5_. The corresponding zero Kelvin voltage curves, relative to a lithium metal reference electrode, are shown in (c) and (d). The experimental voltage curves for the E[2 × *∞*] (R-Nb_2_O_5_) and E_1_[4 × 4] (N-Nb_2_O_5_) forms of Nb_2_O_5_ were measured by Parui et al.^37^ and Lian et al.,^42^ respectively.

Figures 3a and b show the formation energies of Li_*x*_Nb_2_O_5_ in the E[2 × *∞*] and E_1_[4 × 4] structures, respectively, as a function of Li concentration. Figures 3c and d show the corresponding voltages at zero Kelvin relative to a metallic lithium reference electrode. At zero Kelvin, only the lowest energy Li-vacancy orderings that reside on the convex hull (i.e., common tangent construction applied to formation energies) are thermodynamically stable. These orderings are referred to as the ground states. The voltage relative to metallic Li is linearly related to the Li chemical potential of the electrode, which at zero Kelvin is linearly related to the slope of the convex hull as a function of Li concentration.^64^ Each step in the voltage profile corresponds to the voltage window in which a particular ordered ground state is stable, while each plateau corresponds to the voltage at which one ground state transitions to an adjacent ground state.^64^ While the formation energies of a large number of Li arrangements within the two host structures were calculated (987 in E[2 × *∞*] and 1265 in E_1_[4 × 4]), it is not possible to perform an exhaustive enumeration of all possible orderings. As a result, the true ground states may not have been determined for each host.

Figure 3c and d show experimental voltage curves for Li_*x*_Nb_2_O_5_ having the E[2 × *∞*] structure as measured by Parui et al.^37^ and for Li_*x*_Nb_2_O_5_ having the E_1_[4 × 4] structure as measured by Lian et al.^42^ Li-ions in Wadsley–Roth phases tend to disorder at room temperature and exhibit sloping voltage profiles that are characteristic of solid solutions.^18,45,65^ As is evident in Figure 3c, the voltage curve for the E[2 × *∞*] structure as measured by Parui et al.^37^ overlaps the zero Kelvin voltage between *x* = 0 and 2, but is higher than the calculated voltage above *x* = 2. The measured voltage curve for the E_1_[4 × 4] structure^42^ is substantially higher than the zero Kelvin voltage curve (Figure 3d). A possible explanation for the discrepancies between the measured and calculated voltage curves is that the true Li-vacancy ground state orderings have not been identified. For example, while the energies of 1265 different Li-vacancy arrangements over the interstitial sites of the E_1_[4 × 4] structure have been explicitly calculated, the large unit cell of this structure results in a combinatorial explosion of possible Li-vacancy orderings, even within the primitive unit cell of the host. In spite of these computational limitations, the very large number of configurations considered in this study for both the E[2 × *∞*] and E_1_[4 × 4] forms of Li_*x*_Nb_2_O_5_ should provide invaluable insights about redox mechanisms in these compounds.

### B Ion-Centered Redox at Dilute Li Concentrations

**Figure 4:**
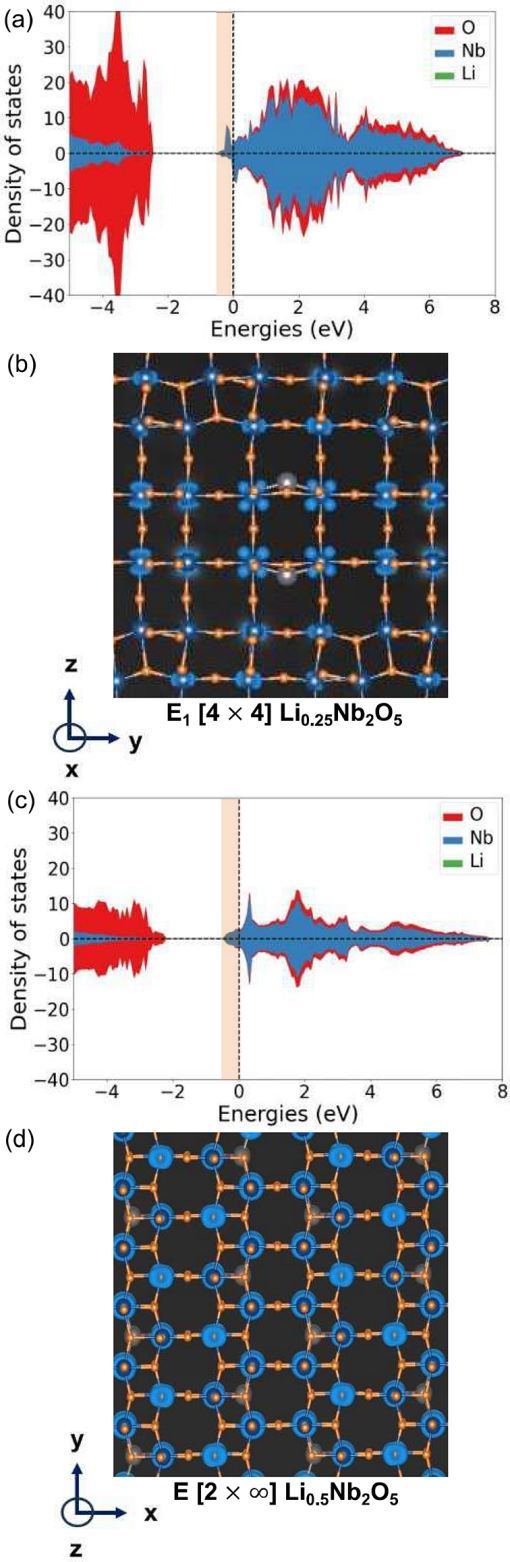
Density of states (a) and charge density (b) for E_1_[4 × 4] Li_0.25_Nb_2_O_5_. The charge density plot is in a plane perpendicular to the -axis, which is parallel to the block length. Density of states (c) and charge density (d) for E[2 × *∞*] Li_0.5_Nb_2_O_5_. The charge density plots are on a plane perpendicular to the -axis. The -axis is again parallel to the block length of the E[2 × *∞*] structure.

The Nb cations of Nb_2_O_5_ are octahedrally coordinated by oxygen and are in their maximum oxidation state of +5, having a d^0^ electron configuration. A large band gap separates the valence bands, comprised primarily of oxygen p states, from empty conduction bands, made up of the Nb derived t_2g_ states (i.e., the Nb 4d_*xy*_, 4d_*yz*_, and 4d_*xz*_ orbitals when octahedrally coordinated by oxygen). The electrons that accompany the inserted Li^+^ ions reduce the Nb^5+^ cations by filling the t_2g_ derived conduction bands. First-principles studies of Nb- and W- containing Wadsley–Roth phases by Koçer et al.^44,52^ showed that the first transition metal cations of Wadsley–Roth phases to reduce are those that reside in corner-sharing octahedra at the center of the *n* × *m* blocks. This was subsequently also predicted in PNb_9_O_25_.^18^Figure 4a and b show the DOS and electron charge density, respectively, of Li_0.25_Nb_2_O_5_ in the E_1_[4 × 4] structure. Upon the insertion of a dilute concentration of Li, the Fermi level moves into the t_2g_ derived bands (Figures 4a). The electronic charge density of the filled t_2g_ states (Figures 4b) shows a high charge density on the Nb of the corner sharing octahedra at the center of the [4 × 4] block. Consistent with previous first-principles calculations of Wadsley–Roth phases with dilute Li concentrations,^44,45,52^ charge density is also evident on the other Nb within the crystal. Note that the DOS of Figure 4a shows a spin up density that is slightly higher than that of the spin down density. This results in a net magnetic moment, which has been observed and predicted in Wadsley–Roth phases containing a dilute concentration of Li.^16,45,52^

Figures 4c and d show similar DOS and electron charge density plots for dilute Li_0.5_Nb_2_O_5_ in the E[2 × *∞*] structure. Due to the very narrow block dimension in one direction, there are no Nb that reside in exclusively corner-sharing octahedra. The electrons donated to the host upon the insertion of a dilute concentration of Li are therefore uniformly distributed over all the Nb cations. In both the E_1_[4 × 4] and E[2 × *∞*] structures, the increase in charge density is localized in atom-centered orbitals.

### C Structural Signatures of Metal-Dimer Redox

While the donation of electrons to the Nb_2_O_5_ host upon the insertion of Li increases the charge density of atom-centered t_2g_ orbitals at dilute Li concentrations, the mechanism of charge accommodation changes at intermediate to high Li concentrations. Similar to predictions for TiNb_2_O_7_,^45^ a metal-dimer redox mechanism involving the bonding states of hybridized t_2g_ orbitals of edge-sharing transition metal cations becomes evident at more concentrated Li concentrations within the E_1_[4 × 4] and the E[2 × *∞*] structures of Nb_2_O_5_.

**Figure 5:**
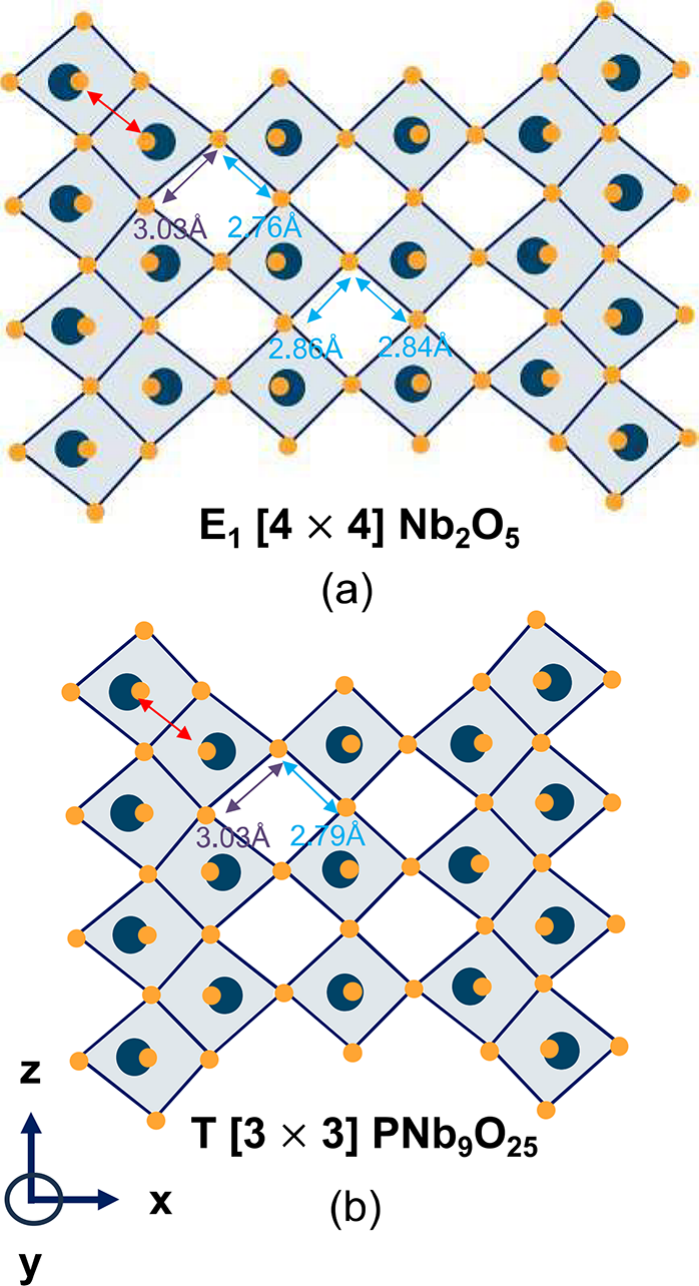
(a) A slice of the E_1_[4 × 4] Wadsley–Roth crystal structure along the block length. The orange circles are oxygen, and the blue circles are transition metal cations. The edge-sharing MO_6_ octahedra along the shear boundaries are highly distorted due to the strong electrostatic repulsion between edge-sharing metal cations. This leads to highly distorted window sites that can host Li-ions. (b) The T[3 × 3] structure of PNb_9_O_25_ undergoes similar distortions along the shear boundaries.

One signature of the onset of the metal-dimer redox mechanism is a shortening of the distance between pairs of edge-sharing metal cations. In pristine Wadsley–Roth phases (without Li) the electrostatic repulsion between edge-sharing transition metal cations in their fully oxidized state induces a significant off-centering and an increase in metal–metal distances.^15,52,66^ This is evident in Figure 5a, which shows a vertical cut through the Nb_2_O_5_ E_1_[4 × 4] structure. As shown by the red arrow in Figure 5, the Nb in the edge-sharing octahedra along the shear boundaries are displaced away from each other, leading not only to an increase in the distance between pairs of edge-sharing transition metal cations, but also significant distortions of the coordinating octahedra of oxygen ions. Figure 5b shows similar increases in metal–metal distances along the edges of the PNb_9_O_25_ structure, which adopts the T[3 × 3] Wadsley–Roth structure.

**Figure 6:**
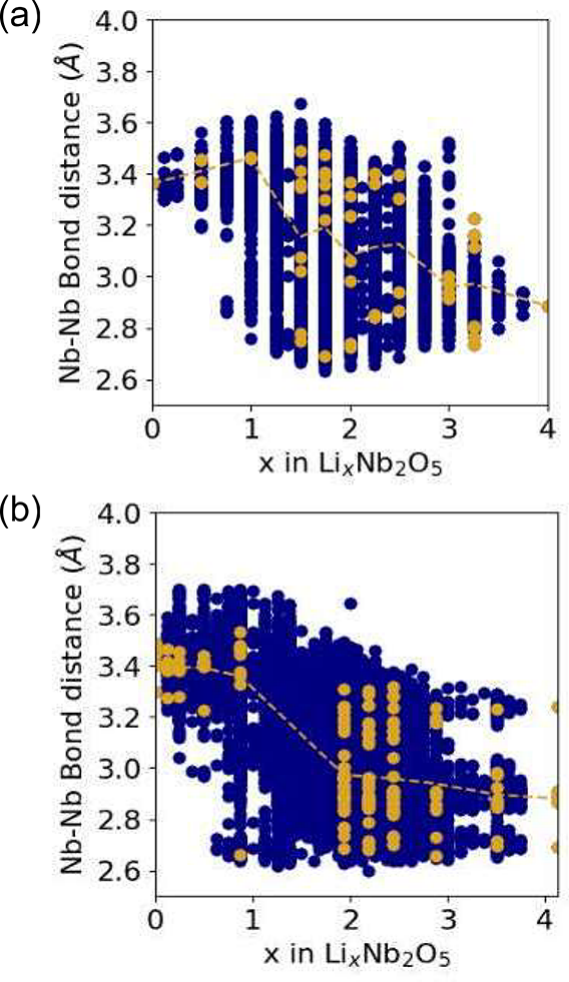
Bond lengths between edge-sharing Nb cations of (a) E[2 × *∞*] Nb_2_O_5_ and (b) E_1_[4 × 4] Nb_2_O_5_ as a function of Li concentration for all structures (blue) and ground state structures (gold) where the gold line indicates the mean bond length for Nb cations in Nb sites in ground state structures. The bond lengths were collected from the fully relaxed DFT calculations of 987 (1265) Li configurations in E[2 × *∞*] (E_1_[4 × 4]) Nb_2_O_5_.

The formation of bonding states between the t_2g_ orbitals of edge-sharing Nb to accommodate electrons donated by inserted Li leads to a contraction of the metal–metal pair distance. Figure 6 collects the pair distances between edge-sharing Nb cations in the E[2 × *∞*] and E_1_[4 × 4] structures of Nb_2_O_5_ as a function of Li concentration. These pair distances were extracted from 987 and 1265 fully relaxed Li-vacancy configurations in the E[2 × *∞*] and E_1_[4 × 4] structures of Nb_2_O_5_, respectively. Figure 6 shows that the distances between a subset of edge-sharing Nb cations undergo a significant contraction (from approximately 3.4 Å to almost 2.6 Å) upon the insertion of 0.5 to 1.5 Li per transition metal. The gold points are metal–metal pair distances of edge-sharing Nb cations in the lowest energy configurations. Since there are multiple edge-sharing metal–metal pairs in each structure, there are multiple pair distances for each lowest energy configuration.

Figure 6 shows that there is a wide distribution of Nb–Nb pair distances, with a spread ranging between 2.6 and 3.7 Å. The Nb–Nb pair distances are sensitive to the Li concentration and the Li-vacancy ordering at a fixed composition. At room temperature, the Li ions are disordered, as manifested by the experimentally measured sloping voltage profiles.^37,42^ Lithium disorder will result in a distribution of Nb–Nb distances with little or no long-range order among the Nb–Nb pairs that form dimers. Hence, structural models obtained with diffraction experiments will reflect an average Nb–Nb bond length. A calculation of the average Nb–Nb bond length at room temperature would require an in-depth statistical mechanics study, which is beyond the scope of this work. The dashed gold lines in Figure 6 show the average Nb–Nb pair distances in the ground state structures as a function of composition. The average decreases gradually as a function of Li composition. The structural models of Li_*x*_Nb_2_O_5_ in the E[2 × *∞*] structure as characterized by Parui et al.^37^ show a similar contraction of the average Nb–Nb bond lengths with increasing Li concentration. For Nb_2_O_5_, they find an average Nb–Nb distance of 3.28 Å, while at *x* = 1 and *x* = 3, the average Nb–Nb distance decreases to 3.15 and 3.13 Å, respectively. We note that, while the calculated average bond distances exhibit a similar decreasing trend, there is a quantitative discrepancy between calculated and experimental values. For example, the calculated Nb–Nb distance in pristine E[2 × *∞*] Nb_2_O_5_ is 3.36 Å, which is larger than the experimental value by approximately 2.5%. In part, this can be attributed by a slight over-prediction of the E[2 × *∞*] Nb_2_O_5_ lattice parameters with DFT-PBE. The calculated lattice parameters are *a* = 4.005, *b* = 3.838, and *c* = 13.012, which are larger than those measured by Parui et al.^37^ (*a* = 3.975, *b* = 3.823, and *c* = 12.706). The *c* lattice parameter is over-predicted by 2.5%, an overestimate that is slightly larger than typical for DFT-PBE.

The formation of metal-dimers has macroscopic consequences. As was shown for Nb- and W-containing Wadsley–Roth phases,^44^ PNb_9_O_25_^18^ and TiNb_2_O_7_,^45^ a contraction in the distances between a subset of edge-sharing cations along the crystallographic shear planes and corners leads to an elongation of the block length and a contraction along the block waist. A useful measure of this dimensional change is a strain order parameter defined as a symmetry-adapted linear combination strain according to^67^
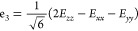
1

The strains are measured within a Cartesian coordinate system with its -axis aligned parallel to the Wadsley–Roth block length, while the - and -axes are in the plane of the *n* × *m* blocks.

**Figure 7:**
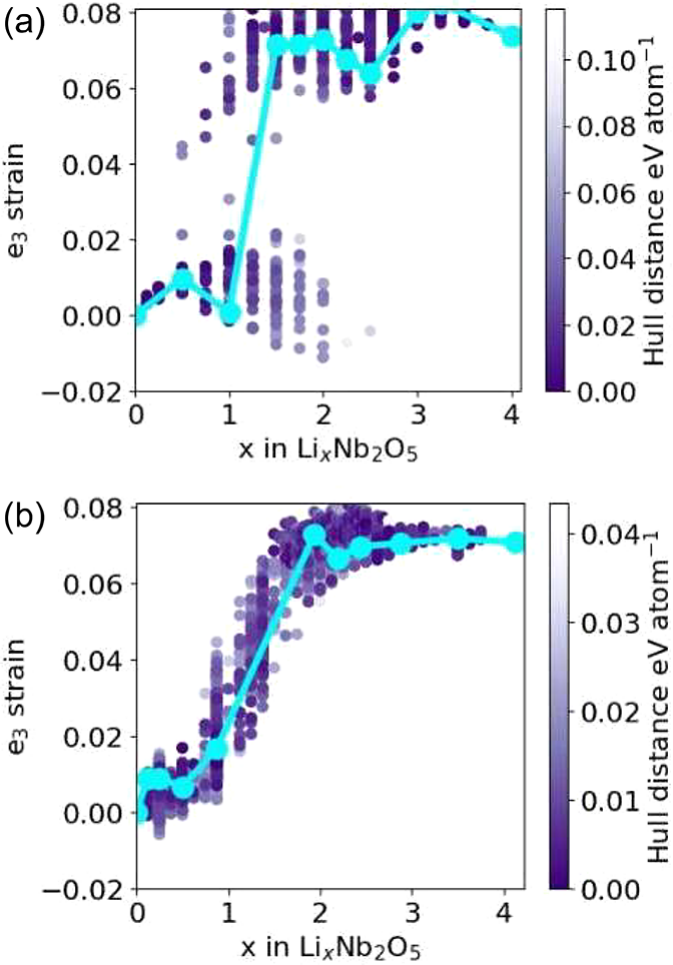
Evolution of e_3_ strain as a function of the Li concentration for (a) E[2 × *∞*] and (b) E_1_[4 × 4] Li_*x*_Nb_2_O_5_.

Figure 7 collects the e_3_ strain order parameter for each of the fully relaxed Li-vacancy orderings in the E[2 × *∞*] and E_1_[4 × 4] structures of Nb_2_O_5_. There is an abrupt increase in e_3_ for *x* between 0.5 and 1.5, which coincides with the onset of metal-dimer formation. The transition is especially abrupt for the E[2 × *∞*] structure, but is more smooth and gradual in the E_1_[4 × 4] structure, presumably due to its large diversity in edge-sharing Nb pairs.

### D Electronic Signature of Metal-Dimer Redox

**Figure 8:**
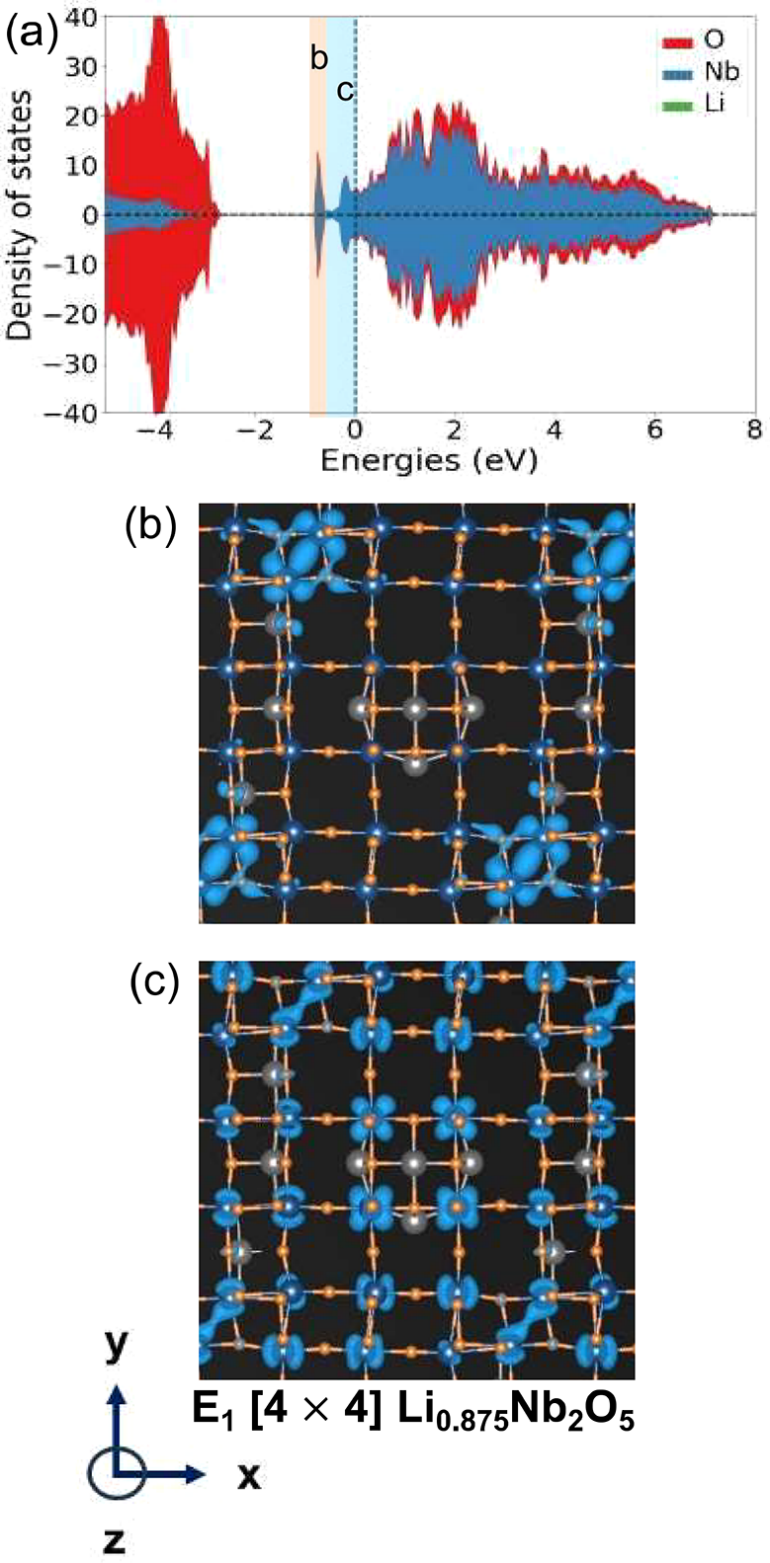
(a) Density of states for the lowest energy E_1_[4 × 4] Li_0.875_Nb_2_O_5_ ordering. (b) The electronic charge density for states with energies highlighted in orange in (a). (c) The electronic charge density for states with energies highlighted in blue in (a).

The bonding states of metal-dimer pairs are highly localized and host both an up and down spin electron. Figure 8a shows the electronic density of states (DOS) of the lowest energy Li ordering within Li_*x*_Nb_2_O_5_ in the E_1_[4 × 4] structure at *x* = 0.875, the first E_1_[4 × 4] structure on the convex hull to undergo metal-dimer redox. The Fermi level in Figure 8a is denoted by the vertical dashed line. The DOS below ≈ −3 eV corresponds to oxygen-dominated levels, while the DOS above ≈ −1 eV is derived primarily from Nb t_2g_ orbitals. Especially striking in the DOS is a sharp peak below the Fermi level at −1 eV due to the formation of a metal–metal dimer. Figure 8b plots the electronic charge density corresponding to this peak. The charge density adopts the characteristic shape of two d_*xy*_ orbitals centered on neighboring Nb cations with a clear enhancement of charge density between the pair of cations. The cations reside in octahedra of oxygen that share a common edge similar to the schematic of Figure 2. The DOS of Figure 8a shows that there is an equal number of up spin and down spin electrons occupying the states corresponding to the metal–metal dimer. The metal-dimer pair consists of Nb cations that share the largest number of edges with other Nb cations. These cations are coordinated by four other cations in a high oxidation state and therefore experience a large driving force to undergo redox to a lower oxidation state. The formation of the metal-dimer bond leads to a contraction of the distance of the pair of Nb cations to approximately 2.6 Å, which is significantly smaller than the average pair distance of 3.4 Å between the other edge-sharing Nb pairs that do not form metal–metal bonds. The charge density due to the remaining t_2g_ derived states above the peak and extending up to the Fermi level is shown in Figure 8c. These states are more uniformly distributed over all the Nb cations within the crystal. Since these states have a sizable density at the Fermi level, they are expected to contribute to electron conduction.

**Figure 9:**
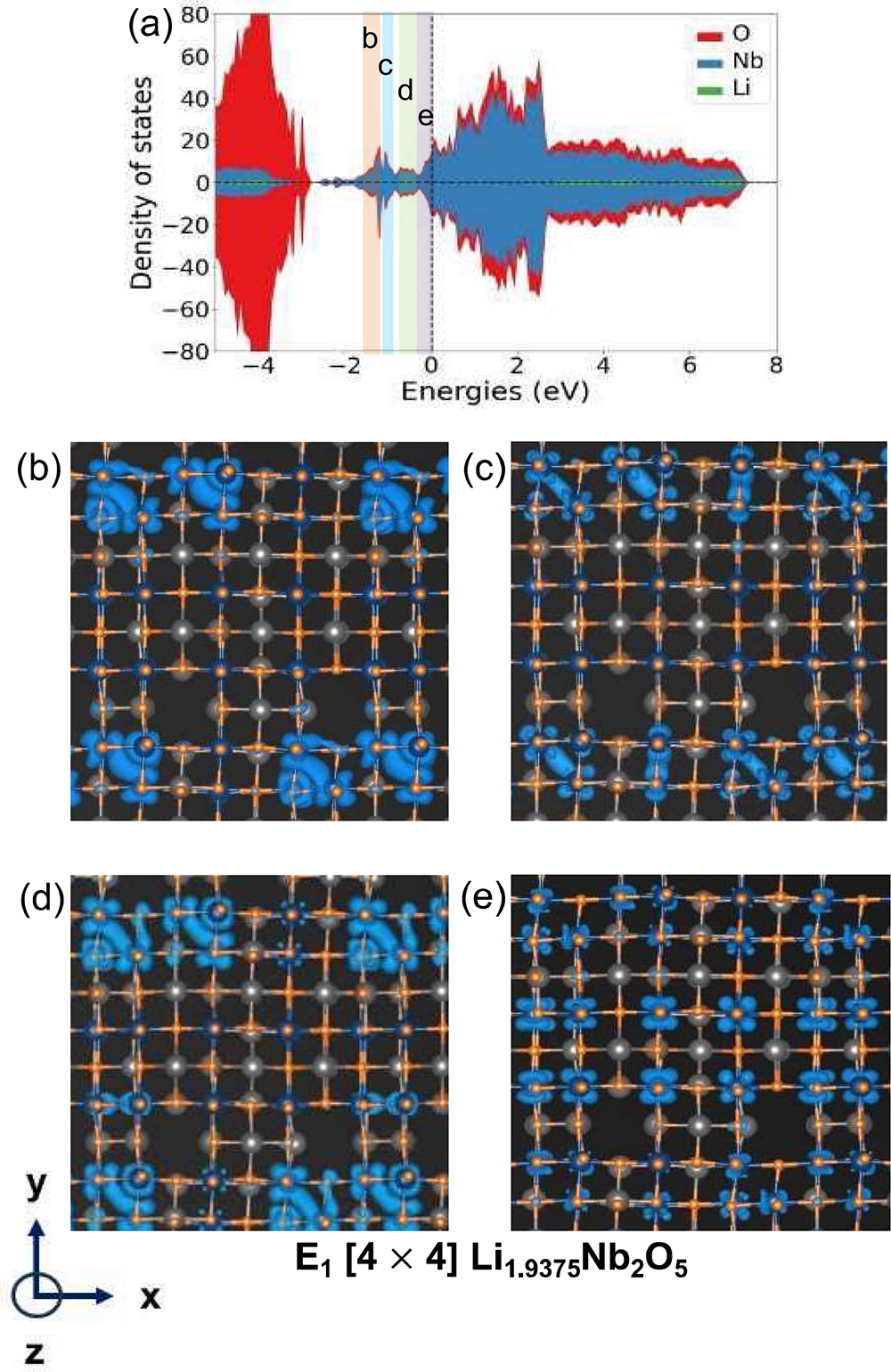
(a) The total density of states of Li_1.9375_Nb_2_O_5_ in the E_1_[4 × 4] structure. The charge densities due to electron states within different energy intervals are shown in (b), (c), and (d). All charge densities are shown in a plane perpendicular to the -axis (which is parallel to the block axis).

Figure 9a shows the DOS for the next E_1_[4 × 4] convex hull structure at *x* = 1.9375. In this structure, the distances between several more edge-sharing Nb pairs have contracted from a mean of 3.36 Å at *x* = 0.875 to 2.97 Å at *x* = 1.9375 in Li_*x*_Nb_2_O_5_. The DOS of Figure 9a shows multiple localized peaks below the Fermi level. The charge densities associated with several of the well-localized peaks are shown in Figure 9b, c, and d. The peaks correspond to localized charge densities between neighboring Nb cations and indicate the formation of metal–metal bonding states that are filled by spin up and spin down electrons. The Nb cations that form dimer bonds share 4, 3, and 2 edges with neighboring Nb. Figure 9e shows the electronic charge density of states with energies close to the Fermi level. The states close to the Fermi level are more centered on Nb cations and are also more uniformly distributed throughout the crystal.

**Figure 10:**
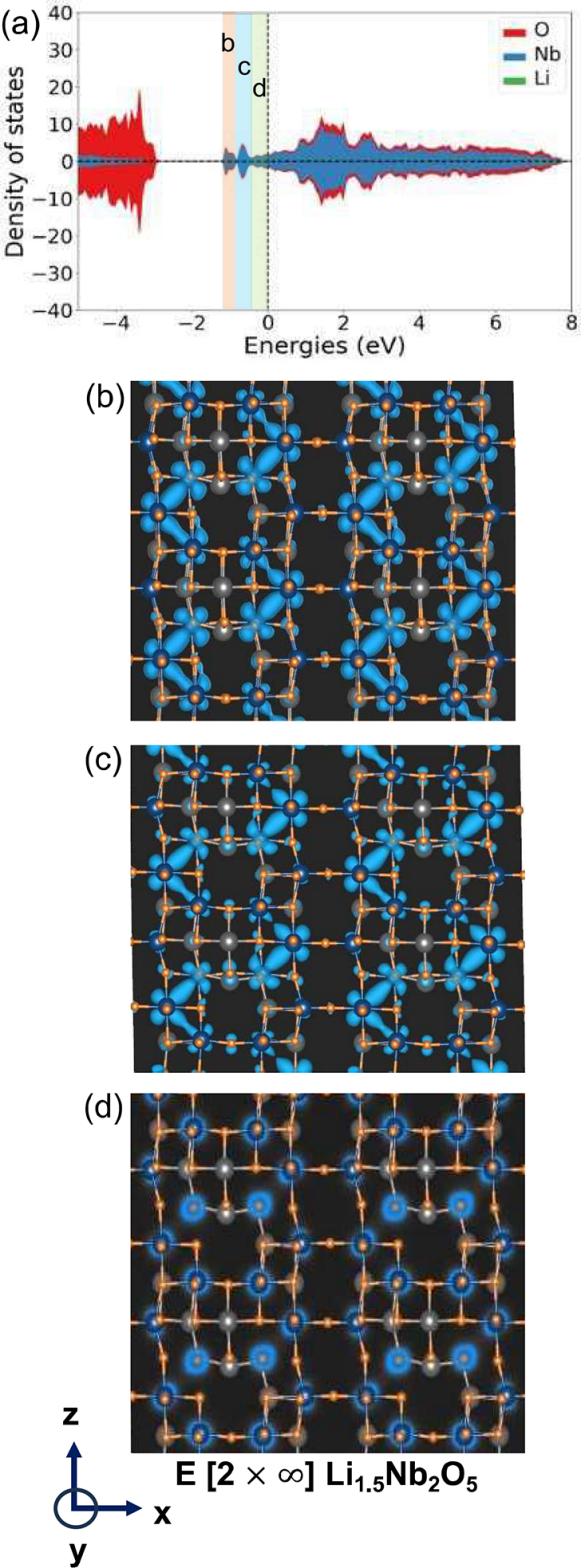
(a) The total density of states of E[2 × *∞*] Li_1.5_Nb_2_O_5_. Charge densities for electronic states with energies in different intervals are shown in (b), (c), and (d). The charge density plots are shown in a plane that is perpendicular to the -axis. The block axis is parallel to the -axis.

The E[2 × *∞*] structure has less diversity in the environments surrounding edge-sharing Nb pairs. As is clear from Figure 6a, the formation of shortened Nb–Nb distances occurs at a higher Li concentration in E[2 × *∞*] than in E_1_[4 × 4] (Figure 6b). Metal-dimer formation in the E[2 × *∞*] structure only starts at *x* = 1.5 as is evident by the emergence of several peaks below the Fermi level in the DOS of Figure 10a. The charge density associated with these peaks, shown in Figure 10b and c, are more uniformly distributed over a large number of metal–metal pairs. In contrast to the E_1_[4 × 4] structure, in which some Nb cations are coordinated by 4 cations and others by 3 or 2 Nb cations, all the Nb cations of E[2 × *∞*] reside in an identical environment, being coordinated by 2 edge-sharing Nb cations. Hence, the onset of Nb–Nb bonding states is more uniform, but occurs at a higher Li concentration, as more electrons are required to fill a larger number of symmetrically equivalent bonding states.

### E Octahedral Distortions Due to Redox Mechanisms

The pristine Wadsley–Roth phases containing early transition metal cations such as M = Ti, Nb, and/or W have highly distorted MO_6_ octahedra.^15,44,68−73^ There are two primary driving forces for these large octahedral distortions. The first is due to the d^0^ electronic configuration of the metal cations of most Wadsley–Roth phases, which makes early transition metals susceptible to an off-centering second-order Jahn–Teller distortion.^44^ The second arises from the high density of edge-sharing octahedra along the shear boundaries of Wadsley–Roth phases. The transition metal cations residing in edge-sharing octahedra experience a strong electrostatic repulsion that results in a sizable off-centering that in turn causes collateral distortions of the coordinating octahedra of oxygen ions.^15^

The redox reactions that accompany Li insertion into Wadsley–Roth phases affect both driving forces of octahedral distortions. The atom-centric redox mechanism at dilute Li concentrations, which results in a filling of the empty d orbitals on early transition metal cations, removes the susceptibility for a second-order Jahn–Teller distortion. The formation of metal–metal bonds at higher Li concentrations leads to a contraction of the edge-sharing Nb–Nb bond distances. The cations forming metal–metal bonds are pulled to the center of their coordinating oxygen octahedra, thereby undoing the strong distortions of the surrounding oxygen ions.

**Figure 11:**
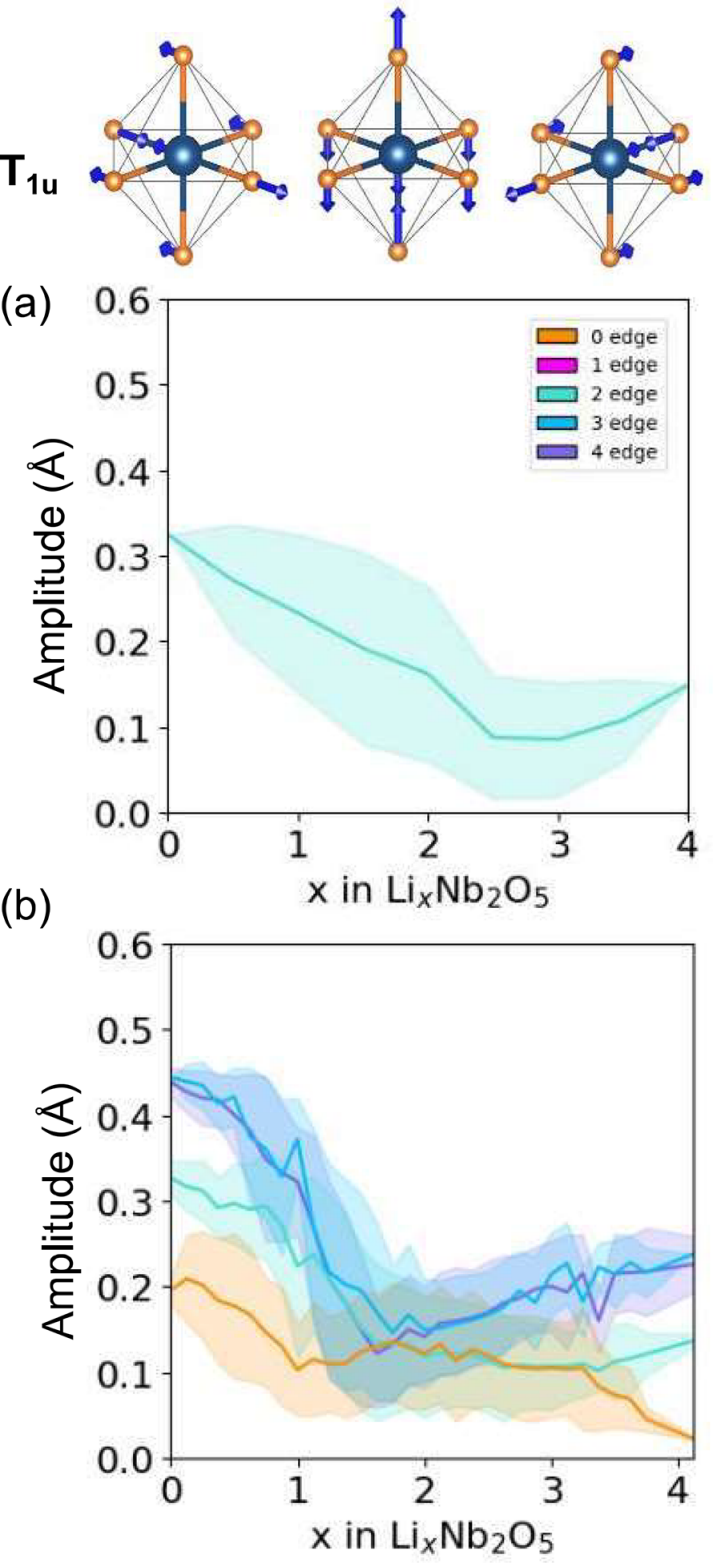
(a) T_1u_ collective octahedral distortion modes that measure the degree of cation off-centering relative to the coordinating oxygen ions. The T_1u_ distortion modes for NbO_6_ octahedra in the of E[2 × *∞*] (b) and E_1_[4 × 4] (c) forms of Li_*x*_Nb_2_O_5_ as a function of lithium concentration.

There are two classes of symmetry-adapted collective displacement modes of a NbO_6_ octahedron that capture the octahedral distortions of pristine Wadsley–Roth phases.^15,45^ The first class, described by the three orthogonal displacement modes shown in Figure 11a, corresponds to a cation off-centering and is a measure of the second-order Jahn–Teller distortion. The amplitudes of this distortion mode were collected for each NbO_6_ octahedron in each fully relaxed Li-vacancy configuration in the E[2 × *∞*] and E_1_[4 × 4] forms of Li_*x*_Nb_2_O_5_. The displacement vectors of the seven ions of an NbO_6_ octahedron undergoing a symmetry-adapted collective distortion mode can be collected in a 7 × 3 = 21 dimensional vector that is normalized to have a length of 1. The amplitudes plotted in Figure 11b and c, with units of Å, are the coefficients of a decomposition of the fully relaxed displacement field of a NbO_6_ octahedron onto the symmetry-adapted collective displacement mode of Figure 11a, as described by Saber et al.^45^Figures 11b and c show the average amplitude and a one-standard-deviation spread around the average of this off-centering distortion mode as a function of the Li concentration for the E[2 × *∞*] and E_1_[4 × 4] structures, respectively. The off-centering amplitude is large in the pristine phases and at dilute Li concentrations. The amplitude of a particular octahedron is also very sensitive to the number of edges that it shares with neighboring octahedra. The corner-sharing octahedra at the center of the 4 × 4 blocks of E_1_[4 × 4] (orange curve) has the smallest off-centering amplitude. The octahedra that share three or four edges in the E_1_[4 × 4] structure have the largest amplitude. The E[2 × *∞*] structure consists of only one type of NbO_6_ octahedron, each sharing only two edges. The variation of the off-centering amplitude of octahedra that share only two edges with Li concentration is very similar in both the E_1_[4 × 4] and E[2 × *∞*] forms of Nb_2_O_5_.

**Figure 12:**
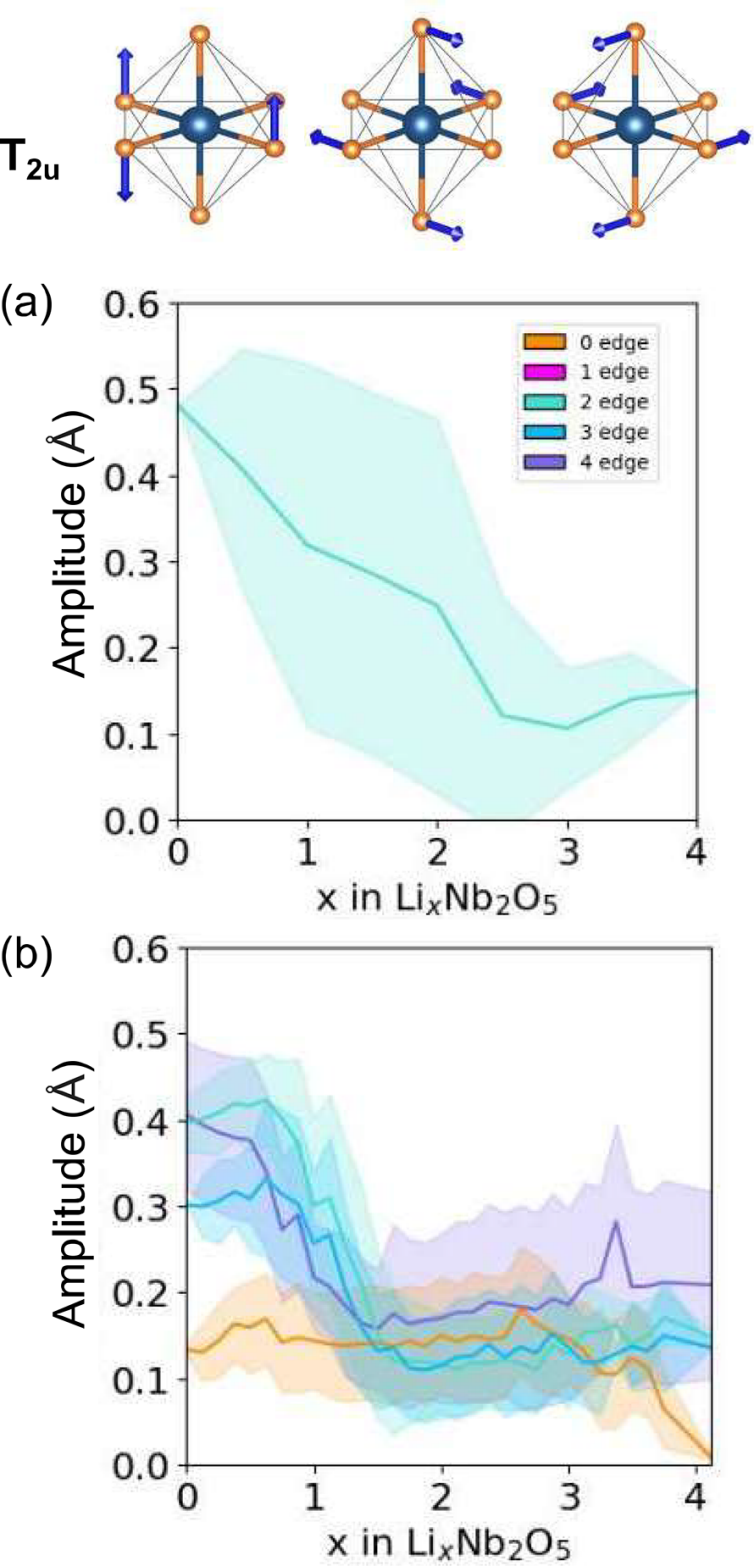
(a) T_2u_ collective octahedral distortion modes that measure symmetry-breaking deformations of the coordinating oxygen ions of Nb cations. T_2u_ distortion modes for NbO_6_ octahedra in the E[2 × *∞*] (b) and E_1_[4 × 4] (c) forms of Li_*x*_Nb_2_O_5_ as a function of lithium concentration.

A second class of octahedral distortion modes captures the deformations of the oxygen ions surrounding edge-sharing Nb cations. The three orthogonal displacement modes that span this class of distortions are shown in Figure 12a. Figures 12b and c show the average and one-standard-deviation spread for the E[2 × *∞*] and E_1_[4 × 4] structures. Here as well, the amplitudes of this distortion mode are very large at dilute Li concentrations and very sensitive to the number of edges that a particular octahedron shares with neighboring octahedra. The octahedra that share edges with neighboring octahedra undergo an abrupt reduction in their distortion amplitude between *x* = 1 and 2, which coincides with the onset of metal–metal dimer formation. The behavior is very similar to that of other Wadsley–Roth phases^18,45^ and early transition metal compounds such as anatase TiO_2_.^74^

## IV Discussion

The open crystal structures of Wadsley–Roth phases makes them attractive electrode materials for Li-ion batteries. They can intercalate large quantities of Li-ions and can sustain rapid electrochemical cycling.^19,24,32,75−77^ Unfortunately, the Li intercalation voltages of promising anode materials having a Wadsley–Roth crystal structure, such as TiNb_2_O_7_,^1^ PNb_9_O_25_,^17^ and ternary Nb–W oxides (e.g., Nb_16_W_5_O_55_ and Nb_18_W_16_O_93_),^2^ are too high to be commercially competitive. A major objective in the design of new Wadsley–Roth phases for anode applications is, therefore, to identify chemistries and structures that have lower voltages than current candidates. This requires a detailed understanding of redox mechanisms and the chemical and structural factors that affect them.

Our first-principles study of the electronic properties of Li_*x*_Nb_2_O_5_ having the E_1_[4 × 4] and E[2 × *∞*] structures predict the occurrence of two distinct redox mechanisms and reinforce similar conclusions of previous studies on related materials.^18,44,45^ At dilute Li concentrations, electrons fill ion-centered transition metal t_2g_ orbitals.^15,18,44^ This occurs more or less uniformly; however, as shown by Koçer et al.,^44,52^ there is a slight preference for the transition metals in the corner-sharing octahedra at the center of the blocks. At higher Li concentrations, the redox mechanism is predicted to change,^45^ involving the low-energy bonding states of metal–metal dimers. The t_2g_ orbitals of edge-sharing transition metal cations hybridize to form bonding states that can accommodate electrons donated by Li (Figure 2). From our examination of redox mechanisms in two Nb_2_O_5_ polymorphs, we have found that metal–metal bond formation, responsible for significant strain in TiNb_2_O_7_,^45^ also occurs in Nb_2_O_5_, suggesting a universality of the metal–metal bond redox mechanism in Wadsley–Roth phases. The dimer redox mechanism belongs to a class of redox processes that occur on extended molecular orbitals.^48−51,78^

The formation of metal–metal dimers has structural consequences, as it results in a shortening of the distance between edge-sharing cations. This in turn affects the lattice parameters and leads to shape changes of the crystal with changes in the Li concentration. The metal–metal dimers form first between transition metal cations that have the highest number of edge-sharing neighbors. These cations experience the strongest electrostatic interactions with other cations in high oxidation states, producing a large driving force to undergo redox and thereby reduce their oxidation state. The E_1_[4 × 4] structure has more flexibility in accommodating the formation of metal-dimers than the more ordered E[2 × *∞*] structure. This is because the E_1_[4 × 4] structure has a wider diversity of local environments, containing NbO_6_ octahedra that share zero, two, three, and four edges with neighboring octahedra. The E[2 × *∞*] structure, in contrast, has only one octahedral environment, with each NbO_6_ octahedron sharing exactly two edges with neighboring octahedra. As a result, E_1_[4 × 4] Nb_2_O_5_ forms metal–metal bonding at a lower lithium concentration than E[2 × *∞*] Nb_2_O_5_.

**Figure 13:**
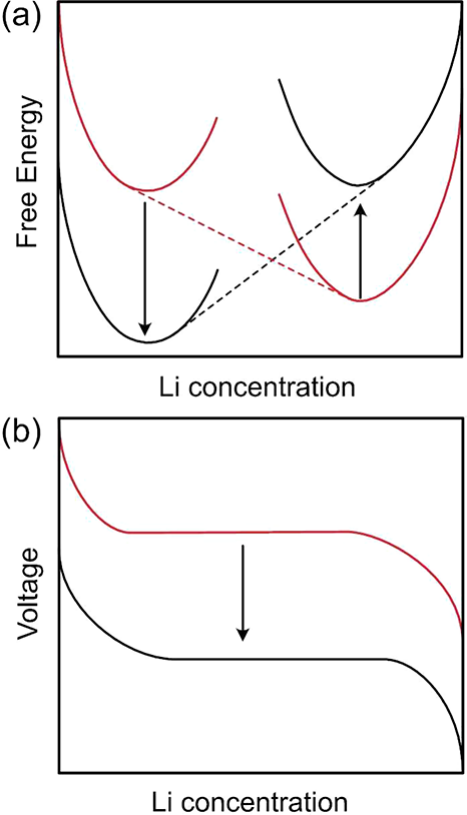
Voltage of a Wadsley–Roth transition metal oxide can be tailored through crystallographic and chemical modifications that alter the free energy of the host as a function of Li concentration (a). The voltage (b) is related to the slope of the free energy curve with respect to Li concentration. The voltage can be decreased by decreasing the free energy at dilute Li concentrations and increasing the free energy at higher Li concentrations.

The results of this study and those of prior studies suggest guidelines with which the structural and chemical diversity of Wadsley–Roth phases can be exploited to tailor the voltage of early transition metal oxides. The voltage of an electrode, as measured relative to a metallic Li reference electrode, is related to the difference in the Li chemical potential of the electrode, , and the chemical potential of metallic Li, , according to the Nernst equation  (where *e* refers to the charge of an electron and where the chemical potentials are in units of eV).^64^ The Li chemical potential in turn is related to the derivative of the Gibbs free energy of the electrode material with respect to concentration. Figure 13 schematically shows two ways in which the voltage of an electrode material can be reduced. One is to lower the free energy of the host at low Li concentrations, while the second is to increase the free energy at high Li concentrations.

In order to lower the voltage of Wadsley–Roth phases, new structures and chemistries must be identified that have a low free energy in the pristine state and/or upon the addition of a dilute concentration of Li-ions, but an unfavorable free energy at higher Li concentrations. This can be realized by using the most stable Wadsley–Roth polymorph for a given transition metal oxide composition. Low-energy Wadsley–Roth phases have structures that make them sufficiently flexible to accommodate octahedral distortions and that have cations arranged to minimize electrostatic interactions between edge-sharing octahedra (i.e., the highest oxidation state cations should be segregated to the center of the blocks occupying corner-sharing octahedra^15,44^).

It may not always be possible to use the most stable pristine Wadsley–Roth crystal structure for a particular transition metal oxide composition if the structure inhibits fast charge/discharge kinetics. In that scenario, it is desirable to identify a structure that has a large number of favorable Li sites at dilute concentrations. This will ensure that the free energy decreases rapidly upon the insertion of a dilute concentration of Li-ions. While Li tends to first fill pyramidal sites along the shear boundaries, the window sites are closer to the transition metal cations that undergo redox at dilute Li concentrations, and thereby more favorable from electrostatic considerations. The window sites tend to be highly distorted in pristine Wadsley–Roth phases due the large distortions of the fully oxidized and edge-sharing MO_6_ octahedra.^15,45^ Wadsley–Roth structures with larger block sizes, however, contain more square window sites that are less distorted and thereby more favorable for Li occupancy.^18,45^ This is evident in Figure 5a, which shows the distortions of the square planar sites of pristine E_1_[4 × 4] Nb_2_O_5_. The square planar sites at the center of the block are less distorted than those along the shear-boundaries. The square planar sites of Wadsley–Roth phases with smaller blocks, such as TiNb_2_O_7_, which has the E_1_[3 × 3] structure, and PNb_9_O_25_, which has the T[3 × 3] structure, are highly distorted due to their close proximity to the shear boundaries and are therefore not favorable for Li occupancy at dilute Li concentrations (Figure 5b).^18,45^ Dilute Li in these compounds tend to fill the pyramidal sites along the shear boundaries, which are further away from the electron donated to the Nb at the center of the block.^18,45^

The voltage can be further lowered by suppressing metal–metal bond formation at intermediate to high Li concentrations. At nondilute Li concentrations, the results of this study and prior work^18,45^ predict that the redox mechanism of Wadsley–Roth phases transitions from an atom-centered mechanism to one that relies on the formation of metal–metal bonds. Structures that are more resistant to shortened metal–metal bonds will have a higher free energy when this redox mechanism becomes active. A stiff crystalline backbone will require the conversion of a portion of the chemical free energy gain of forming metal–metal dimers into elastic energy, thereby lowering the voltage. This, however, may lead to undesirable hysteresis in the voltage profile.^79^ An alternative is to identify alloying elements that do not favor metal–metal dimer formation and also segregate to the edge-sharing octahedra at the shear boundaries. Wadsley–Roth phases containing Nb, V, and Mo, which are known to readily form metal–metal bonds,^46−49^ should then be alloyed with elements that have a lower oxidation state. Alloying elements having a low maximum oxidation state will tend to segregate to the edge-sharing octahedra along the crystallographic shear planes^15^ and thereby relegate the metal-dimer formers such as Nb, V, and Mo to corner-sharing octahedra where they are unable to form metal–metal bonds. The addition of alloying elements with lower oxidation states than Nb or W will stabilize Wadsley–Roth crystal structures with smaller block sizes or Wadsley–Roth phases with partially reduced transition metals to preserve charge neutrality.^15^ The suppression of metal-dimer redox will lead to a reliance on less favorable, atom-centered, redox mechanisms, and thereby a lowering of the voltage at higher Li concentrations.

## V Conclusion

We have performed a first-principles investigation of redox mechanisms accompanying Li insertion in two very distinct Wadsley–Roth crystal structures of Nb_2_O_5_. Consistent with previous first-principles studies of other Wadsley–Roth phases,^18,44,45^ we identify two redox mechanisms: (i) an atom-centered redox mechanism that dominates at dilute Li concentrations and (ii) a dimer redox mechanism at intermediate to high Li concentrations whereby the bonding states of edge-sharing metal-dimers accommodate the electrons donated by Li to the host. The dimer redox mechanism leads to the shortening of metal–metal pair distances. This makes the mechanism highly dependent on the elastic flexibility of the host crystal structure. The results of this study suggest design principles with which the electrochemical properties of Wadsley–Roth phases can be tailored through chemical and structural modifications of the host.

## Supporting Information Available

Projected density of states for the high lithium concentration E_1_[4 × 4] Nb_2_O_5_ ground state ordering; symmetry-adapted displacement mode definitions; displacement mode amplitudes as a function of lithium concentration for E[2 × *∞*] Li*_x_*Nb_2_O_5_ and E_1_[4 × 4] Li*_x_*Nb_2_O_5_ (PDF)
