# Supplementary material for: Redox Mechanisms upon the Lithiation of Wadsley–Roth Phases
Source: Inorg Chem. 2024 Jun 4;63(24):11041–52. doi: 10.1021/acs.inorgchem.4c00603 (PMC11186016; doi:10.1021/acs.inorgchem.4c00603)
Supplement: Supplementary file 1 — ic4c00603_si_001.pdf [file ic4c00603_si_001.pdf]

Supporting information
Redox mechanisms upon the lithiation of Wadsley–Roth phases

Muna Saber[1] and Anton Van der Ven[2,*]

[1]*Department of Chemical Engineering, University of California, Santa Barbara, Santa Barbara, California 93106, United States*

[2]*Materials Department, University of California, Santa Barbara, Santa Barbara, California 93106, United States*

(Dated: May 15, 2024)

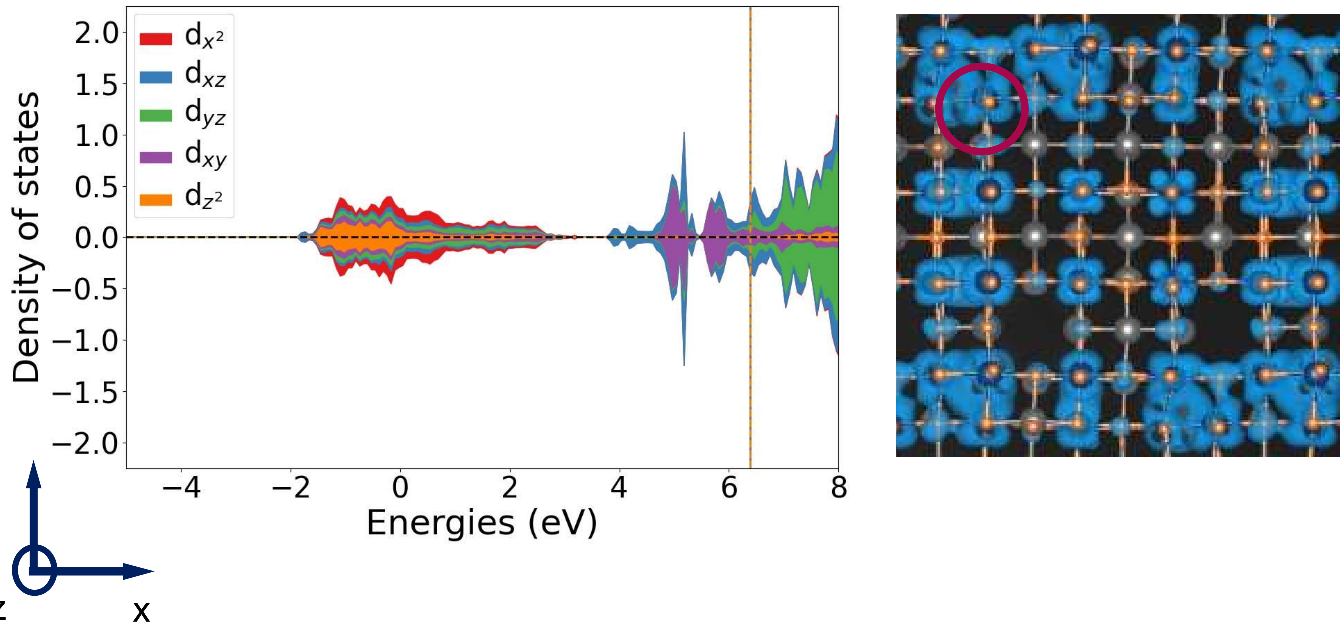

FIG. S1. The projected density of states for the 1st Nb Site in $Li_{1.9375}Nb_2O_5$, an Nb site within an octahedra that shares 4 edges at the corner of the 4 by 4 block.

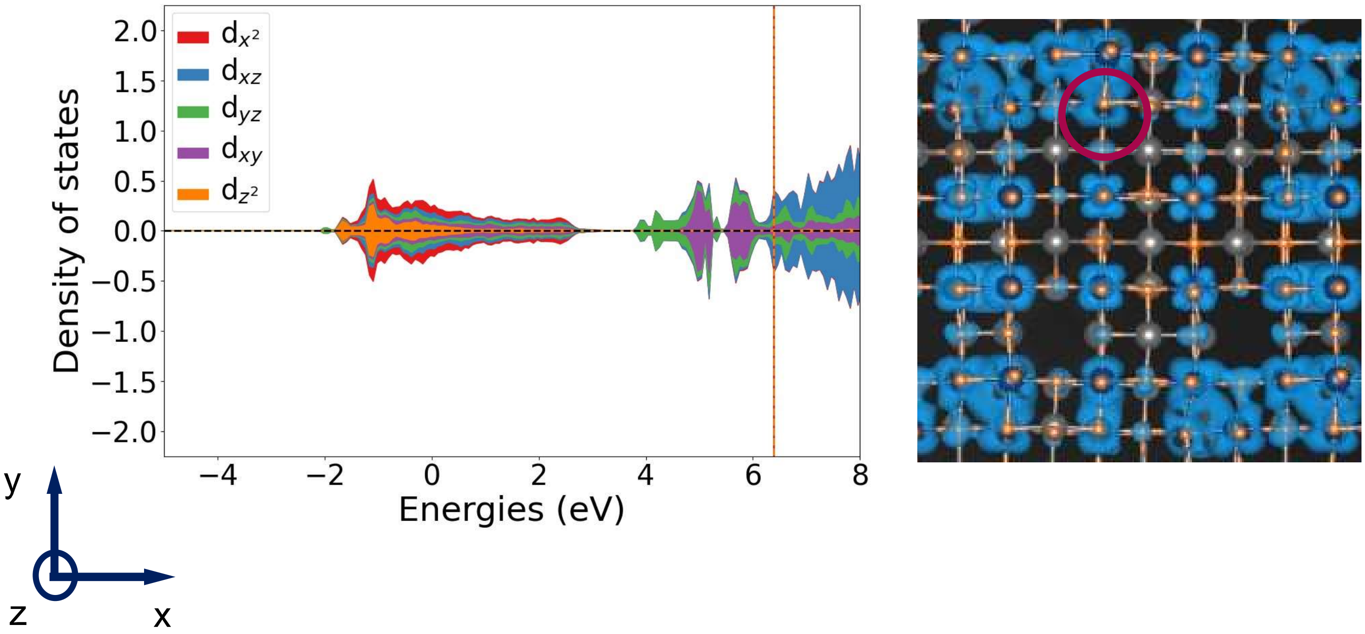

FIG. S2. The projected density of states for the 2nd Nb Site in $Li_{1.9375}Nb_2O_5$, an Nb site within an octahedra that shares 3 edges at the corner of the 4 by 4 block.

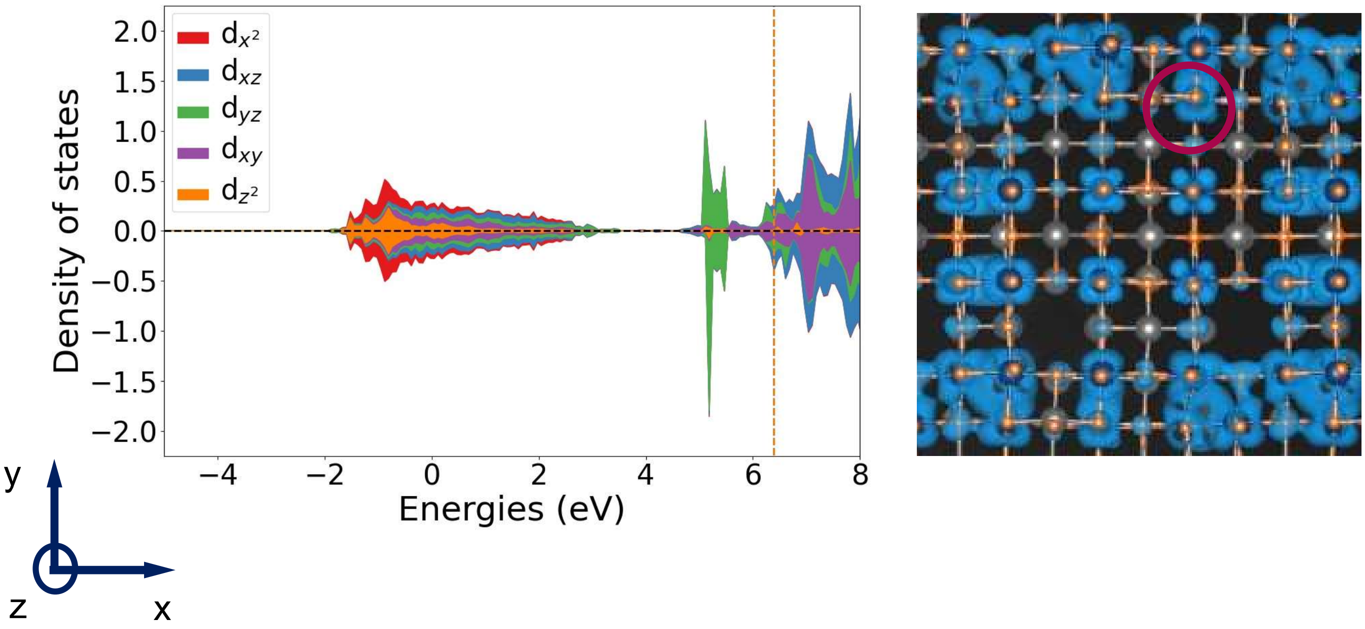

FIG. S3. The projected density of states for the 3rd Nb Site in $Li_{1.9375}Nb_2O_5$, an Nb site within an octahedra that shares 2 edges at the top of the 4 by 4 block.

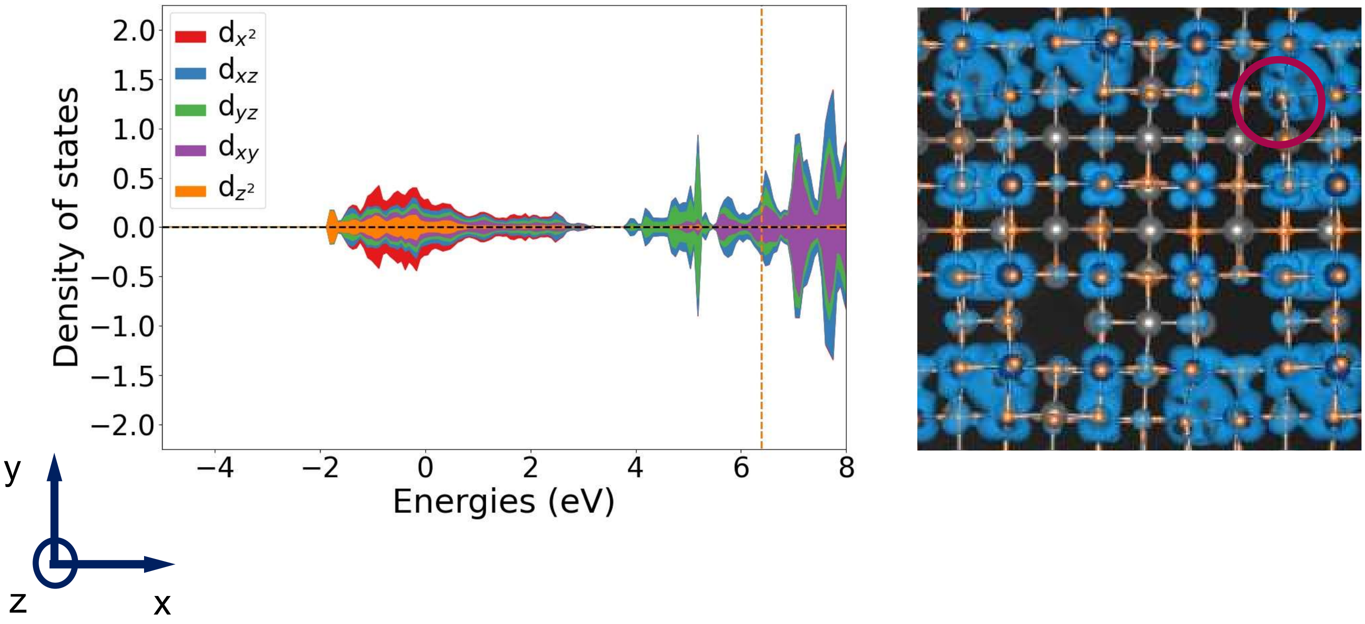

FIG. S4. The projected density of states for the 4th Nb Site in $Li_{1.9375}Nb_2O_5$, an Nb site within an octahedra that shares 4 edges at the corner of the 4 by 4 block.

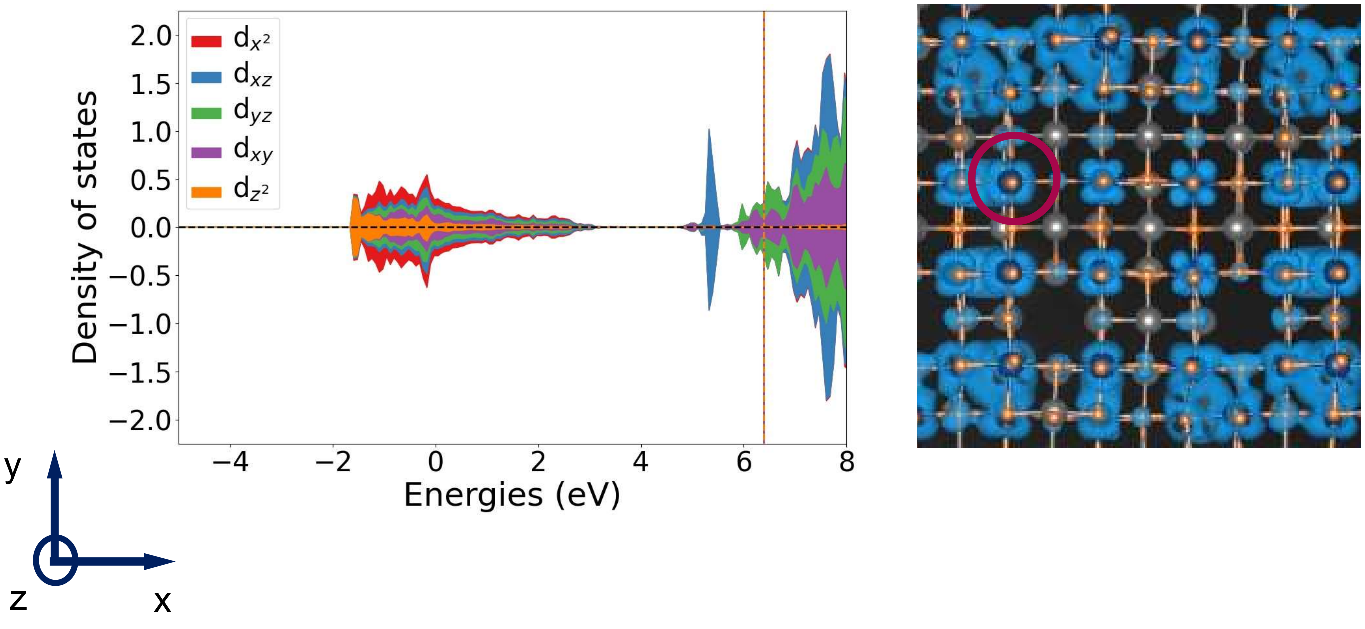

FIG. S5. The projected density of states for the 5th Nb Site in $Li_{1.9375}Nb_2O_5$, an Nb site within an octahedra that shares 2 edges at the side of the 4 by 4 block.

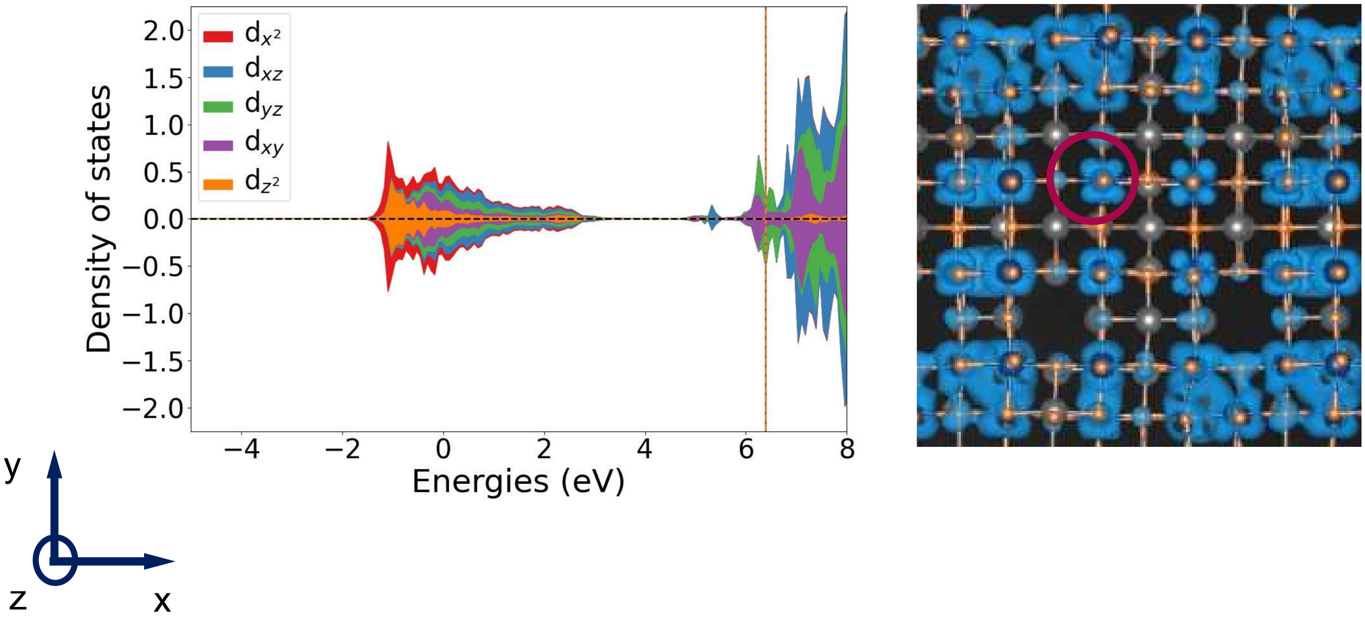

FIG. S6. The projected density of states for the 6th Nb Site in $Li_{1.9375}Nb_2O_5$, an Nb site within an octahedra that shares 0 edges at the center of the 4 by 4 block.

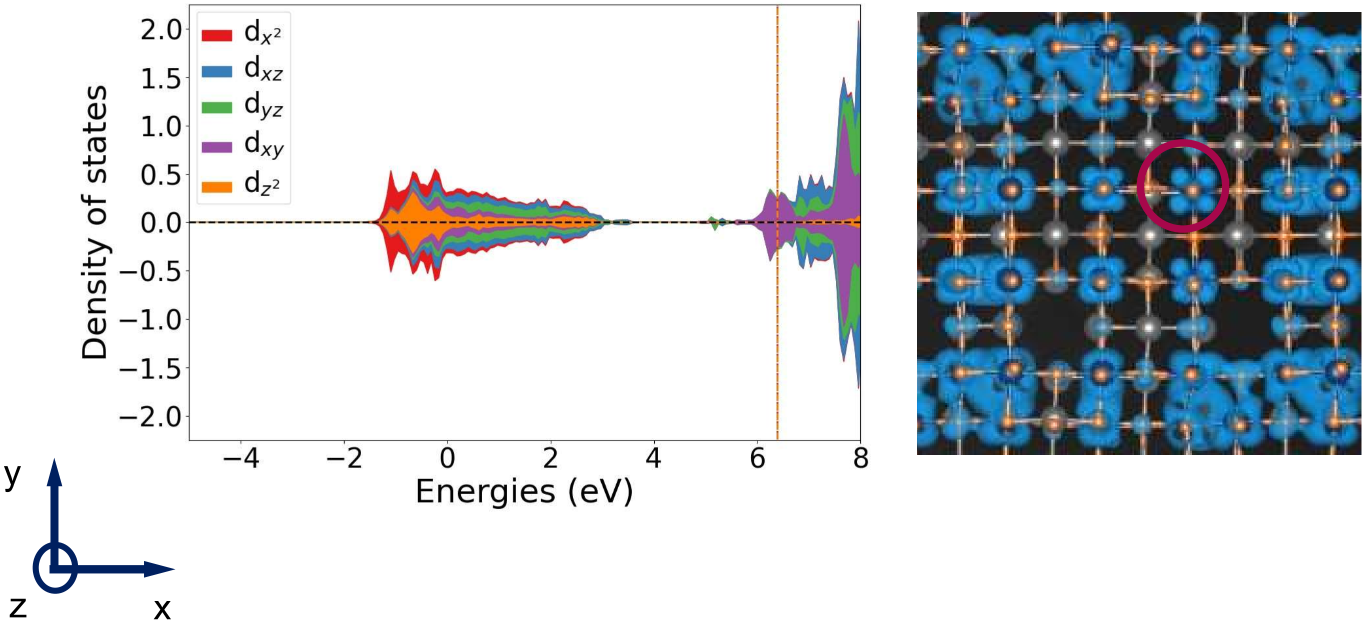

FIG. S7. The projected density of states for the 7th Nb Site in $Li_{1.9375}Nb_2O_5$, an Nb site within an octahedra that shares 0 edges at the center of the 4 by 4 block.

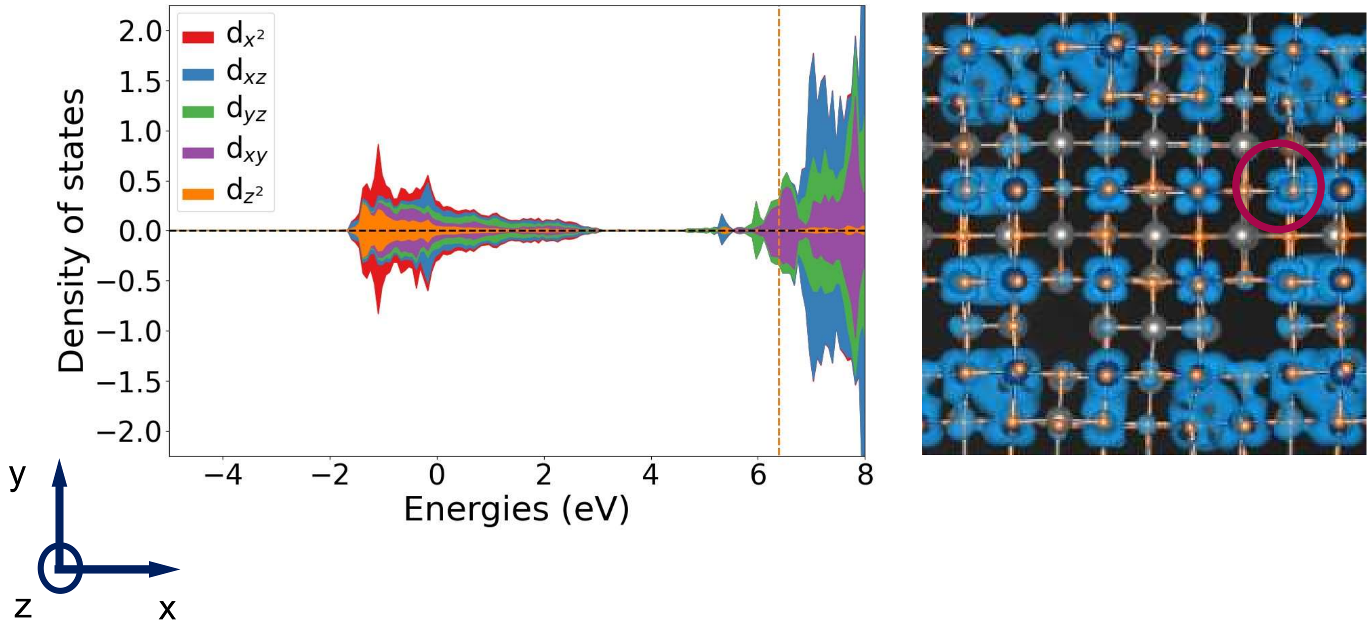

FIG. S8. The projected density of states for the 8th Nb Site in $Li_{1.9375}Nb_2O_5$, an Nb site within an octahedra that shares 2 edges at the side of the 4 by 4 block.

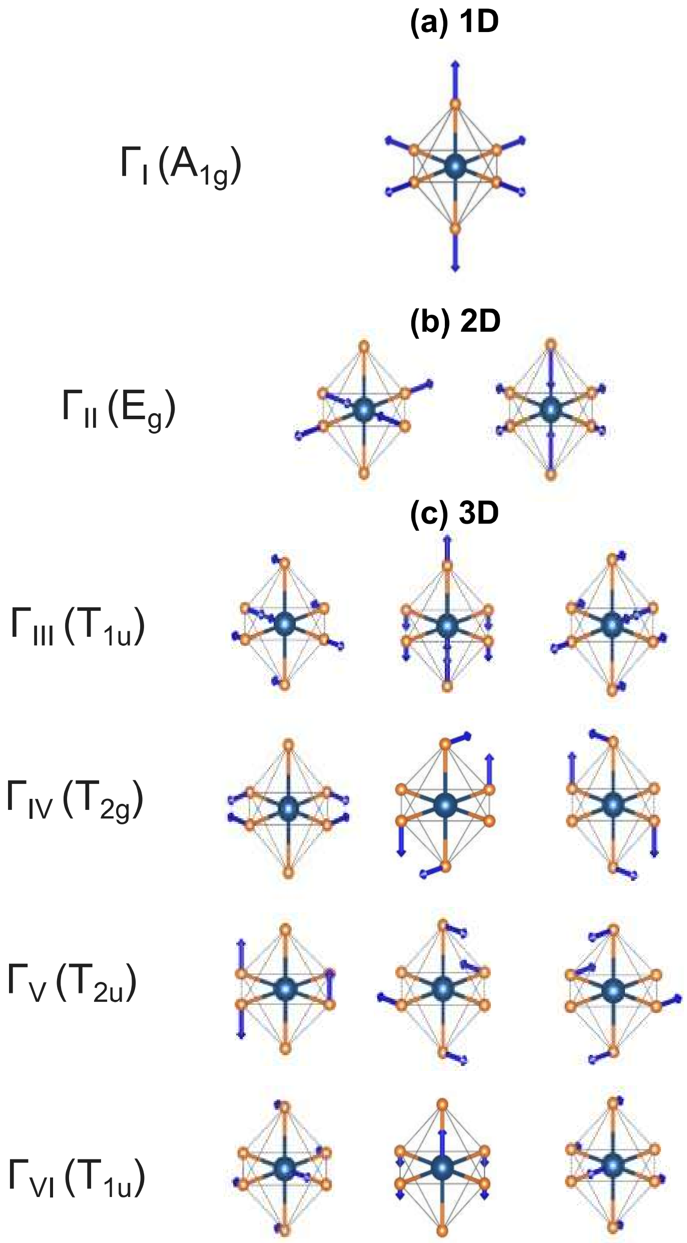

FIG. S9. Symmetry adapted displacement modes for a reference MO_6 octahedron. Adapted from Saber and Reynolds et al.[1]

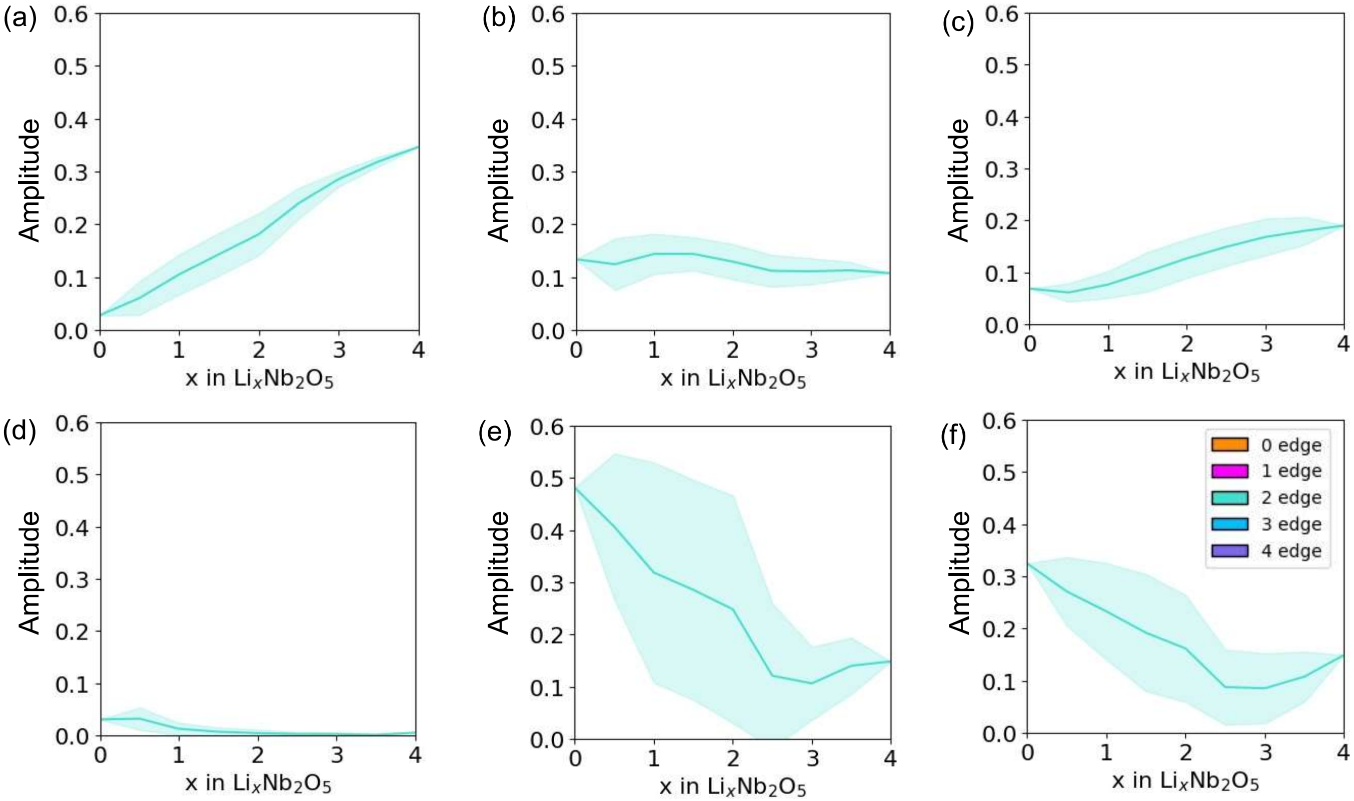

FIG. S10. The displacement modes for all NbO_6 octahedra all Li-vacancy orderings within the primitive cell of E$[2 \times \infty]$ Nb_2O_5 as a function of Li concentration. Distortions are organized by irreducible representation: (a) Γ_1 corresponding to volumetric deformations with point group A_{1g}, (b) Γ_2 corresponding to tetragonal deformations with point group E_g, (c) Γ_3 corresponding to 3D deformations with point group T_{1u}, (d) Γ_4 corresponding to 3D deformations with point group T_{2g}, (e) Γ_5 corresponding to 3D deformations with point group T_{2u}, (f) Γ_6 corresponding to Second Order Jahn-Teller distortions with point group T_{1u}.

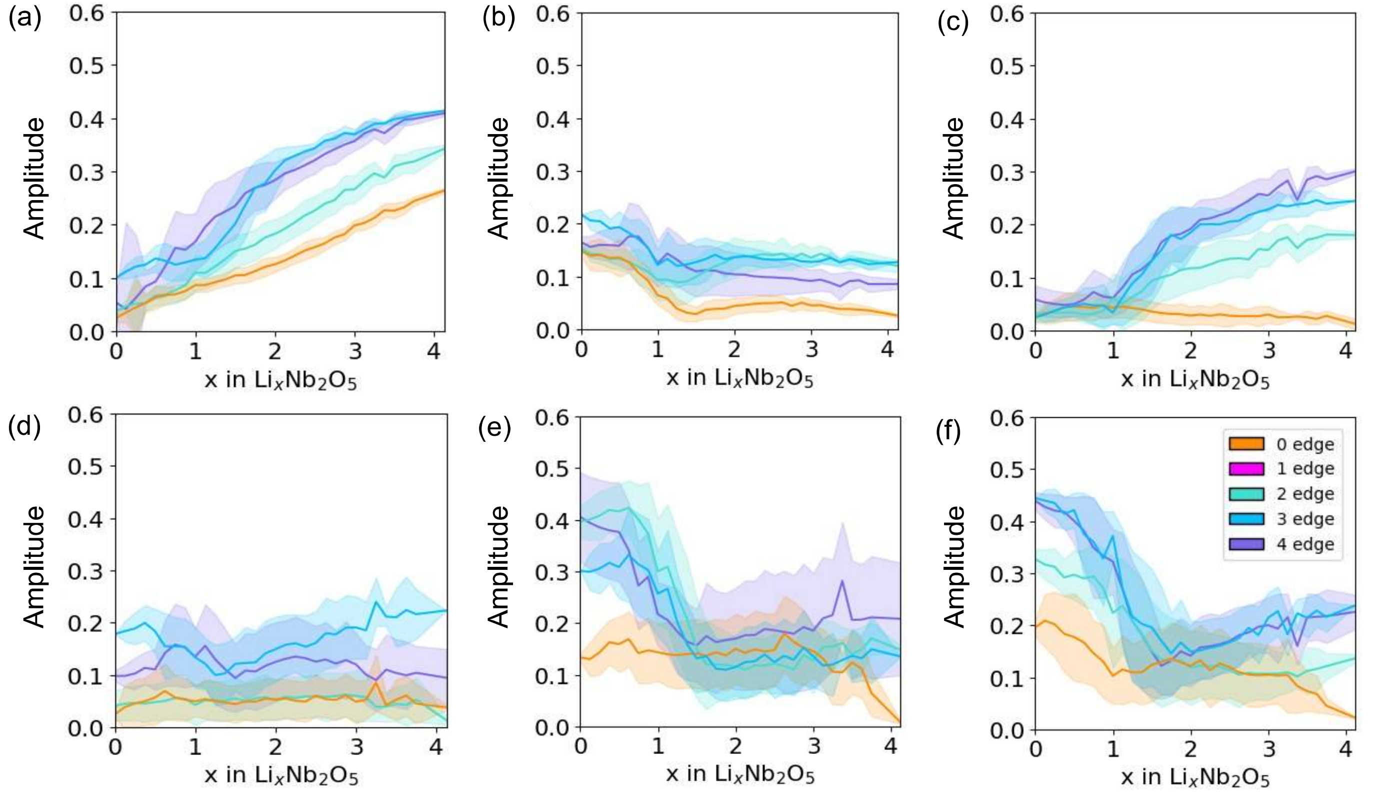

FIG. S11. The displacement modes for all NbO_6 octahedra all Li-vacancy orderings within the primitive cell of $E_1[4 \times 4]$ Nb_2O_5 as a function of Li concentration. Distortions are organized by irreducible representation: (a) Γ_1 corresponding to volumetric deformations with point group A_{1g}, (b) Γ_2 corresponding to tetragonal deformations with point group E_g, (c) Γ_3 corresponding to 3D deformations with point group T_{1u}, (d) Γ_4 corresponding to 3D deformations with point group T_{2g}, (e) Γ_5 corresponding to 3D deformations with point group T_{2u}, (f) Γ_6 corresponding to Second Order Jahn-Teller distortions with point group T_{1u}.

REFERENCES

1. Saber, M., Reynolds, C., Li, J., Pollock, T. M. & Van der Ven, A. Chemical and structural factors affecting the stability of Wadsley–Roth block phases. *Inorganic Chemistry* **62,** 17317–17332 (2023).
